# Honey Bee Viromes from Beekeeping Operations Experiencing High Losses in 2022–2023

**DOI:** 10.3390/v18030334

**Published:** 2026-03-09

**Authors:** Boone H. Jones, Taylor Reams, Lauren Jonas, Brandon K. Hopkins, Michelle L. Flenniken

**Affiliations:** 1 Department of Plant Sciences and Plant Pathology, Montana State University, Bozeman, MT 59717, USA; 2 Department of Microbiology and Immunology, Montana State University, Bozeman, MT 59717, USA; 3 Pollinator Health Center, Montana State University, Bozeman, MT 59717, USA; 4 Department of Entomology, Washington State University, Pullman, WA 99163, USAbhopkins@wsu.edu (B.K.H.)

**Keywords:** honey bee, *Apis mellifera*, honey bee colony losses, colony death, honey bee virome, honey bee virus, partitivirus, Hubei partitivirus, acute bee paralysis virus (ABPV), Lake Sinai virus (LSV)

## Abstract

Recent high annual losses of honey bee (*Apis mellifera*) colonies, averaging 40% in the United States from 2008 to 2025, are concerning for beekeepers, growers, policy makers, and scientists. Viruses, the most abundant group of honey bee pathogens, impact honey bee fitness and contribute to colony losses. Several studies have utilized next-generation sequencing (NGS) technologies to discover new honey beeinfecting viruses and expand our understanding of the honey bee virome. Herein, we examined the viromes of honey bees obtained from longitudinally monitored, commercially managed colonies that experienced population decline (average ~44%) during the 2022–2023 beekeeping season. We hypothesized new viruses or virus genome variants may be associated with these declines. To test this hypothesis, we sequenced RNA obtained from virus-augmented honey bee samples from representative colonies managed by four beekeeping operations in California. We discovered three undescribed partitivirus-like sequences that were prevalent and abundant in all beekeeping operations, a new Lake Sinai virus, and a sequence variant of acute bee paralysis virus. In addition, we re-sequenced the genomes of 16 previously characterized bee and/or *Varroa destructor* mite infecting viruses and two previously described, but not well-characterized, partitivirus-like sequences (i.e., Apis mellifera associated partiti-like virus 1 and Hubeipartiti-like virus 34). Virus abundance was greater in libraries representing colonies that died during the monitoring period.

## 1. Introduction

Metagenomic sequencing projects have expanded our understanding of the viral landscapes of many species and facilitated virus discovery. Lower sequencing costs and the use of sample preparation protocols that enrich for viral nucleic acids have also promoted the discovery of new viruses [1,2,3,4,5]. Reflecting these advances in next-generation sequencing (NGS) technologies and decreased costs, the number of viral genomes on NCBI GenBank expanded from 27,091 in 2010 to ~6.8 million in 2021 [6,7,8]. These virus-sequencing efforts have expanded our understanding of the viruses associated with many organisms, including diverse insect species [9,10,11,12]. Insects are the most abundant animals on Earth, accounting for over one million species, while estimates suggest millions more are undescribed [13,14,15]. Insects provide important ecosystem services, including decomposition, nutrient cycling, pest control, and plant pollination [16]. In general, insect abundance and biodiversity have declined in many areas, though the rate and ubiquity of these declines are debated [17,18,19,20].

Most metagenomic studies have focused on humans and economically important plants and animals, leaving the insect virosphere relatively under-described [21]. In 2016, Shi et al. sequenced samples from over 220 invertebrate species (representing more than 60 different genera of insects) and identified 1445 novel RNA virus sequences [22]. In 2020, Wu et al. bioinformatically examined publicly available RNAseq data from 644 insect species and identified 1213 undescribed RNA virus sequences [11]. These sequencing and bioinformatic studies highlight the underexplored diversity of insect viromes.

Honey bees (*Apis mellifera*) are eusocial insects that live in colonies comprised of ~30,000 individuals [23]. Honey bees pollinate over 100 different crop plants in North America and are important both economically and ecologically [24,25,26]. There have been high levels of managed honey bee colony losses in the U.S. and other parts of the world. In the U.S., annual honey bee colony losses averaged 40% from 2008 to 2025 [27,28,29,30,31,32,33,34,35,36,37,38]. Many factors contribute to colony deaths, including chemical exposure, inadequate nutritional resources, and pathogens [39,40,41,42,43]. Viruses are the most abundant group of honey bee pathogens and are associated with colony losses [44,45,46]. Viruses are readily horizontally transmitted between honey bees within densely populated colonies via contact, including trophallaxis and brood rearing [45]. They may also be vertically transmitted from a queen bee (the sole reproductive unit of the colony) to her offspring [47,48,49,50,51,52]. In addition, viruses may be transmitted by the *Varroa destructor* mite which feeds on developing and adult honey bees [53,54]. Virus-contaminated floral resources that are shared by proximate honey bee colonies and sympatric bee species within a geographic region also serve as a hub for transmission [55,56,57,58,59,60].

Several sequencing efforts have been undertaken to characterize the honey bee virome and identify viruses (or virus strains) associated with colony deaths [5,61,62,63,64,65,66]. These studies resulted in the discovery of numerous previously uncharacterized viruses, including the Lake Sinai viruses (LSVs), Apis rhabdovirus 1 and 2 (ARV-1, ARV-2), Andrena-associated bee virus 1 (AnBV1), and Apis mellifera solinvivirus 1 (AmSV1) [5,61,62,63]. Some of these new viruses were detected in other bee species (e.g., AnBV1 in *Andrena* spp.), suggesting the potential for interspecies virus transmission, as demonstrated for some well-characterized bee viruses (e.g., deformed wing virus, DWV, and sacbrood virus, SBV) [57,67,68]. Further analysis revealed that certain viruses occupied broad geographic ranges, with LSVs detected in every continent containing honey bees, and ARV-1 detected in Africa, North America, and Oceania [5,63]. Recent discoveries of uncharacterized virus sequences by NGS suggest the honey bee virome may be underexplored, opening new avenues for research examining pathogenicity, geography, and host ranges of putative new viruses [64,69,70].

In 2022–2023, U.S. honey bee colony losses averaged ~48%, which was greater than in previous years [71]. To investigate the potential association between viruses and poor honey bee colony health, we sequenced representative samples obtained from a 2022–2023 colony-level monitoring project that included four California based migratory commercial beekeeping operations involved in almond pollination [72]. According to almond crop acreage and honey bee colony reports [73,74], approximately 89% of all honey bee colonies in the US were needed for California almond pollination in 2022 [75]. We hypothesized that there may be previously uncharacterized viruses and/or virus strains (sequence variants) associated with colonies experiencing population declines. Therefore, virus augmented RNA was isolated from select samples and sequenced using the Illumina short-read sequencing platform [4,5,76].

Bioinformatic analyses resulted in the discovery of three undescribed partitivirus-like sequences, a new Lake Sinai virus (LSV-9), and a sequence variant of acute bee paralysis virus (ABPV-CA-22). In addition, 16 well-characterized bee and/or *V. destructor* mite viruses were sequenced. We determined that there was a greater abundance of virus sequences in sequencing libraries representing colonies that either experienced population declines or died during the monitoring period, relative to libraries representing colonies that did not experience population declines.

## 2. Materials and Methods

### 2.1. Honey Bee Samples

Honey bee samples were obtained from four different commercial beekeeping operations in California (A–D, Supplementary Materials, Tables S1–S4). Individual colonies were repeatedly assessed and sampled from all operations in either August or September 2022, November 2022, and January 2023 [72]. Additionally, beekeeping operations A and B were sampled in March and April 2023, respectively (Supplementary Materials, Tables S1–S4). Colonies from one Washington beekeeping operation and one Montana beekeeping operation were also sampled for virus detection. Honey bee samples were stored at –80 °C until analyses.

At each sampling event, *Varroa destructor* mite infestation levels were assessed and colony population size, which is a proxy for colony health, was estimated by count of honey bee covered frames [77,78,79]. Four categories were utilized to describe honey bee colony population dynamics. Specifically, colonies with populations that decreased by more than 40% over the monitoring period were considered “high loss” colonies. Colonies that either maintained their initial population or increased in population over the course of the study were considered “healthy” colonies. Colonies with populations that decreased by 40% or more from August/September to November but had an increase from November to January were categorized as “declined/recovered”. Colonies that had no honey bee covered frames remaining prior to the final sampling date were classified as “died during the monitoring period”. Colonies were classified as “high *V. destructor*” if they had three or more mites per 100 bees, while “low *V. destructor*” colonies had fewer than three mites per 100 bees. "Post high *V. destructor"* colonies were defined as those that had previously high *V. destructor* pressure but subsequently had low *V. destructor* pressure.

### 2.2. Honey Bee Sample Preparation for Virus Analysis

Samples with the same categorical descriptions were pooled (e.g., beekeeping operation A, assessment/sampling in September, high loss, and high *V. destructor* pressure) for sample preparation and sequencing. In total, 38 libraries were sequenced.

For each colony-level sample, ten individual adult honey bees were randomly selected for analysis. Based on estimated and empirical data, analysis of ten bees per colony should result in detection of the most prevalent pathogens (i.e., those infecting ~45% of individuals within the colony) [77,80,81]. Honey bees (five per 2 mL microcentrifuge tube) were homogenized in 500 µL of sterile H_2_O using two sterile steel beads (4.5 mm) and a Qiagen TissueLyser (Germantown, MD, USA) for 4 min at 30 Hz. Bee lysates were centrifuged for two minutes at 13,500 rpm to remove debris, and supernatants were combined to yield a colony-level lysate. A volume of 60 µL of supernatant from each colony-level lysate was pooled with lysates from colonies matching the same criteria to generate a “pooled lysate”. The total volume of colony-level lysates pooled varied with the number of samples per grouping, with a range from 60 to 420 µL (representing one to seven colonies) (Supplementary Materials, Table S5).

#### 2.2.1. Nuclease Treatment

To enrich for viral nucleic acids, bee lysates were treated with nucleases to degrade unencapsulated (non-capsid protected) DNA and RNA using 1 µL Benzonase (~250 U) and 8 µL RNAse 1 (80 U) (Thermo Fisher Scientific, Waltham, MA, USA) in a final volume of 500 µL buffer (20 mM Tris, 1 mM MgCl_2_, 100 mM NaCl, pH = 7.6) for 1.5 h at 37 °C. Nucleases were not subjected to a heat deactivation step to prevent denaturation of virions.

#### 2.2.2. RNA Extraction

RNA was extracted from nuclease treated honey bee lysate samples using TRIzol reagent (Thermo Fischer Scientific) according to the manufacturer’s instructions. In brief, 500 µL of TRIzol was added to each sample. Samples were vortexed and incubated at room temperature for 5 min. Next, 150 µL of chloroform was added and each sample was mixed by inverting for 15 s, followed by incubation at room temperature for 2 min. Samples were centrifuged for 15 min at 12,000× *g*. The upper aqueous layer (500 µL) was transferred to a 1.7 mL microcentrifuge tube; 20 µg of glycogen and an equal volume (500 µL) of isopropanol was then added to each sample. Samples were precipitated at −20 °C for 24 h, then centrifuged for 10 min at 12,000× *g* at 4 °C. Supernatants were carefully removed by pipetting, and each pellet was washed three times with 75% ethanol and once with 100% ethanol. Ethanol was removed by decanting, and RNA pellets were air dried before 15 µL of sterile H_2_O was added to each sample. RNA quality and quantity were assessed on a Nanodrop 3000 (Wilmington, DE, USA). Additional detailed methods are included in the Supplementary Materials, Text S1.

#### 2.2.3. RNA Sequencing

RNA (1 µg) for each sample was sent to the Roy J. Carver Center for Biotechnology at the University of Illinois. Since samples were nuclease treated prior to RNA extraction to enrich for viral RNA, libraries were prepared without poly-A selection or ribosomal RNA depletion using a Watchmaker RNA prep kit (Boulder, CO, USA). RNA quality and quantity were assessed using an Agilent 3500 Fragment Analyzer (Santa Clara, CA, USA). Samples were sequenced on an Illumina Novaseq X Plus 25B Flowcell (San Diego, CA, USA) (2 × 150-cycle, per lane) generating ~3.2 billion total reads. A range of 6,560,090 to 10,694,460 paired-end reads (150 bp) were generated for each library, with an average read count of 8,051,730.

#### 2.2.4. cDNA Synthesis

cDNA was synthesized by incubating 2 µg of total RNA, 200 U of Moloney murine leukemia virus reverse transcriptase (MMLV-RT), and 500 ng of random hexamer primers in a 25 µL reaction at 37 °C for 1 h. cDNA was generated from a representative subset of RNA samples representing colony-level lysates (n = 10) to assess virus presence and abundance.

#### 2.2.5. Polymerase Chain Reaction (PCR)

PCR was performed using standard methods to screen individual colony-level lysates for partitivirus sequence presence. Briefly, 2 µL of cDNA was combined with 10 pmol of each forward and reverse primer (Supplementary Materials, Table S6). Amplification was performed using ChoiceTaq polymerase according to the manufacturer’s instructions with the following conditions: 95 °C for 5 min, 95 °C for 30 s, 60 °C for 30 s, 72 °C for 30 s (for a total of 35 cycles), followed by a final elongation step for 5 min at 72 °C. Generated PCR products were analyzed by gel electrophoresis (2% agarose with SYBR safe dye) and visualized using a Syngene U:Genius 3 imaging system. The primers used for partitivirus PCR and quantitative PCRs are listed in the Supplementary Materials, Tables S6 and S7. To further support the de novo-assembled undescribed virus sequences generated during this study, Sanger sequencing of an approximately 500 nt region of AmPVLC1, AmPVLC2, AmPVLR1, LSV-9, ABPV-CA-22, and Southern California dicistrovirus 1 was performed. PCR products were purified using a QIAquick PCR purification kit (Qiagen) according to the manufacturer’s instructions. DNA quantity and quality were assessed using a Nanodrop 3000 prior to Sanger sequencing. In all cases, Sanger sequenced regions shared >99% nucleotide identity to the assembled genome.

#### 2.2.6. Quantitative Polymerase Chain Reaction

Quantitative PCR (qPCR) was used to analyze the abundance of AmPVLC-1, AmPVLC-2, and HPLV-34 at the colony level. qPCR reactions were performed using 2 µL of cDNA as template in triplicate. Reactions contained 1 × ChoiceTaq Mastermix, 0.2 uM of each forward and reverse primer, 1 × SYBR green, and 3 mM MgCl2. Reactions were carried out in 96-well plates using a CFX Connect Real-Time instrument using Maestro software (v 2.3) (Bio-Rad, Hercules, CA, USA) with the following thermo-profile: preincubation at 95 °C for 1 min, followed by 40 cycles of 95 °C for 10 s, 60 °C for 15 s, and 72 °C for 15 s. A final melt curve was generated (with measurements at every half degree) from 65 °C to 95 °C for 5 s. To quantify the abundance of these sequences, plasmid standards (virus-specific qPCR amplicons cloned into TOPO vectors (Thermo Fisher Scientific) were used as templates, with a range of 10^2^ to 10^9^ copies per reaction (Supplementary Materials, Table S7). Reactions without a cDNA template were used as non-template controls (NTC). The specificity of qPCR reactions was verified by melt curve analysis and gel electrophoresis. For each of the colony-level samples, the starting quantity (SQ) for each well with a cDNA template representing 80 ng of total RNA was calculated using the previously generated virus-specific standard curve, and the average SQ of the NTC reaction was subtracted. Sequence abundance was reported as RNA copies (including potential genomes and transcripts) per 2 µg of total RNA. Abundance ranged from ~0 copies to ~3.8 × 10^10^ RNA copies/2 µg of RNA.

### 2.3. Bioinformatic Analysis

#### 2.3.1. Read File Processing

Additional bioinformatic methods and example commands can be found in the Supplementary Materials, Text S1. Read files were downloaded and quality was assessed using FastQC (v.0.12.1) [82]. To remove low quality reads or possible sequencing adaptors, the trimming tool BBDuk (part of the BBtools suite) was used to trim reads (v39.26) [83]. After trimming, reads were aligned to the reference honey bee genome (NCBI Amel_HAv3.1) and the HoloBee nonviral index (an index of honey-bee-associated microorganisms) using HISAT2 [84,85,86,87,88]. Only unmapped reads were retained using samtools (v 1.21) [89]. The resulting unmapped reads bam file was name-sorted using samtools. This sorted bam file was converted to paired-end FastQ files using samtools prior to assembly.

#### 2.3.2. De Novo Assembly and Clustering

Paired-end FastQ files containing unmapped reads were used to de novo assemble virus contigs using SPAdes (v-4.2.0), with the option -rnaviral to specifically assemble RNA viruses from read files [90]. Assembly was performed for each individual sequencing library, generating 38 contig files (one for each library). These individual assemblies were then concatenated, generating a redundant list of contigs from all samples (i.e., duplicates of the same viral genomes). To create a nonredundant list of contigs, CD-HIT was used to cluster sequences with greater than or equal to 95% nucleotide identity, and only contigs longer than 1000 nt were retained. Contigs remaining after clustering (i.e., those sharing less than 95% nucleotide similarity) were treated as unique. Initial assembly generated 152,898 contigs across all sequencing libraries. After clustering, 1825 nonredundant contigs remained.

#### 2.3.3. Identification of Putative Viral Contigs

The generated list of contigs was queried against a local DIAMOND database, generated from NCBI’s viral RefSeq dataset of viral proteins (release 229), in order to identify putative viral contigs [91,92]. To identify putative viral sequences with more distant homology to known viruses, a local hidden Markov model (HMM) profile was created using the virus orthologous group (VOG) database (v 227) [93]. Open reading frames in the assembled contigs were predicted using Prodigal (v 4.3), and the corresponding translated protein sequences were queried against the VOG HMM profile using HMMscan [93,94]. Putative new virus sequences were further characterized using Geneious (v2025.1) [95]. Across the 1825 nonredundant contigs, 457 had homology to viruses by DIAMOND alignment, while two highly abundant contigs did not share homology with viruses by DIAMOND alignment, but did by HMMscan. Putative viral contigs were further analyzed by web interface BLASTn, enabling the removal of false positives and sequences appreciably shorter than the expected genome length. This resulted in a final list of 68 contigs, representing 16 well-characterized honey bee and/or *V. destructor* viruses, eight plant viruses, and 26 undescribed viruses or virus fragments.

#### 2.3.4. Comparison of Virus Genome Variability Across Sequencing Libraries

To assess the variability of virus sequences between individual libraries and ensure the reported viral sequences were most representative, consensus sequences for each putative viral contig were generated for each sequencing library. This was done by trimming and filtering reads from each sequencing library, followed by alignment to the nonredundant viral contigs using HISAT2, generating a library-specific alignment for each contig. Consensus sequences were generated from these alignment files in cases where at least 1000 reads aligned using the Geneious “Generate Consensus Sequence” function (v.2025.1). The consensus sequences for each virus were aligned using MAFFT with default parameters within Geneious [96]. The consensus sequences for each virus across sequencing libraries were very similar, sharing over 99% nucleotide identity in nearly all cases (Supplementary Materials, Table S8).

Additionally, to prevent potential biases of using a single assembled contig sequence for all alignments and verify the assembled sequences from each library were similar to one another, de novo assembly was performed using reads from each sequencing library. This generated an assembly unique to each library. Contigs representing common bee infecting viruses (i.e., ARV-1, ABPV, BQCV, DWV-A, DWV-B, and SBV) assembled from individual libraries were aligned to one another using MAFFT with default settings. This strategy showed that de novo-assembled viral contigs from each unique sequencing library were similar, sharing >97% nucleotide identity in all cases. Thus, the virus sequences reported here were similar across libraries representing a broad geographic range and read quantification was not biased (as in, reads across libraries were similar enough to the reported sequence to not be unmapped).

#### 2.3.5. Generation of Virus Consensus Sequences

To generate a global consensus sequence representative of all sequencing libraries, reads from each library were independently aligned to the previously assembled nonredundant virus sequences. Each library alignment file (.bam) was merged to generate a global bam using the samtools merge command. A pileup file of all variants was generated using the bcftools mpileup command. Variants were called using the bcftools call command. This generated a global variant call file (.vcf) representing mapped reads across libraries. A consensus sequence was generated from this global variant call file using the bcftools consensus command. To minimize ambiguities, the -H 1 option was used to call the majority allele at each position.

#### 2.3.6. Contig Quantification and Normalization

Relative contig abundances were estimated by aligning the trimmed FastQ read files to the nonredundant list of putative viral contigs with HISAT2. These alignment files were compressed to .bam files and unaligned reads were discarded using samtools. These resulting .bam files were sorted using samtools. To compare virus read abundance across samples, raw read counts for each virus were converted to fragment per kilobase gene per million reads (FPKM). Read counts of each trimmed FastQ file were generated using the Seqkit stats command [97]. Top contigs with homology to known viruses were further characterized using Geneious to assess sequence similarities and predict open reading frames (ORFs). To characterize virus prevalence, a library was considered “positive” for a given virus if 1000 or more reads aligned.

#### 2.3.7. Relaxation of HISAT2 Alignment Parameters

Nonredundant viral contigs were used to create a HISAT2 index, against which reads for each sequencing library were aligned to generate coverage maps and consensus sequences. Due to the highly variable nature of RNA virus populations, HISAT2 alignment was performed with more relaxed parameters (-L, 0, −0.4) to permit a greater proportion of mismatches during alignment (~6–8 mismatches for a 150 nt read, or approximately 94.7% to 96% identity) and more reliably capture all viral reads. By testing alignment parameters, we determinedd that this HISAT2 alignment settingcaptured contigs that were not aligned using default settings (in some instances, ~10% of contigs that did not align under default parameters did align using the relaxed parameter) (Supplementary Materials, Table S9). Further relaxing of alignment parameters did not appreciably increase the proportion of aligned contigs.

#### 2.3.8. Assessment of Read Mapping Specificity

To assess the specificity of read quantification by alignment to the DWV-A and DWV-B sequences, as well as the LSV-2 and LSV-3 sequence variants, alignment was performed using HISAT-2 with either single- or multi-mapping enabled. Differences in read counts for DWV-A, DWV-B, and the LSV variants, using single- or multi-mapping were negligible indicating reads were not aligning to both DWV-A and DWV-B or to multiple LSV variants (Supplementary Materials, Table S10).

## 3. Results

### 3.1. Honey Bee Colony-Level Samples Obtained from Four California Beekeeping Operations That Experienced High Losses

**Figure 1:**
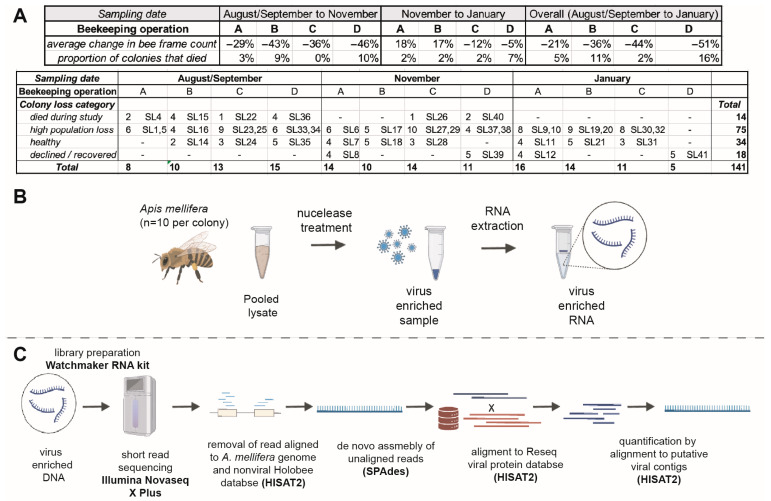
Honey bee colony-level samples, lab processing, and bioinformatic steps to characterize honey bee viromes. (**A**) Honey bee samples were obtained from four California based beekeeping operations. Colony level population change and proportion of colonies that died varied by operation and sample date. Pooled sequencing libraries included RNA from a total of 141 honey bee samples obtained from 67 colonies, including 14 from colonies that died during the monitoring period, 75 from colonies with high population loss, 34 from colonies that either maintained their initial population or increased in population, and 18 from colonies with populations that decreased and then recovered (Supplementary Materials, Tables S1–S4). (**B**) Honey bee samples were homogenized in an aqueous buffer prior to pooling by beekeeping operation, sample date, and categorical assignments. Samples were nuclease treated to degrade unencapsulated nucleic acids, enriching for virion encapsidated nucleic acids, prior to RNA extraction. (**C**) Sequencing libraries were prepared from virus enriched RNA and sequenced. Short-read sequencing data was filtered for quality, the adapter sequences were trimmed, and reads were aligned to the *A. mellifera* genome and Holobee microbe index. Aligned reads were removed from subsequent analyses to facilitate virus discovery. Remaining reads were de novo assembled, and contiguous sequences (contigs) were aligned to the virus RefSeq protein database to identify putative viral contigs. Sequence data was aligned to putative viral contigs to assess abundance, and relative abundance was calculated as fragments per kilobase per million transcripts (FPKM).

Managed honey bee colonies in the U.S. are typically housed in Langstroth hives that contain vertically oriented wooden frames that support the bee-produced wax hexagonal combs that house brood (eggs, larvae, and pupae) and contain food resources (honey and pollen) [23]. Honey bee colony population size, which serves as a proxy for colony health, is estimated by counting the number of honey bee covered frames within each colony at each assessment [77,78,79]. A total of 195 colonies were monitored and sampled in the four California based beekeeping operations (Supplementary Materials, Tables S1–S4) [72]. Initial samples were obtained from colonies in two of the four beekeeping operations in late August 2022 (operations B and D), whereas sampling of colonies in the other two beekeeping operations was initiated in September 2022 (operations A and C). Subsequent samples were obtained from monitored colonies in all four beekeeping operations in early–mid November 2022 and in late January 2023, with additional sampling of beekeeping operation A in March 2023 and beekeeping operation B in April 2023. In this study, a total of 38 sequencing libraries, representing 141 colony-level samples, were sequenced (Figure 1, Supplementary Materials, Tables S1–S5).

The colonies monitored in this study experienced an average 39% decline in honey bee population from Fall 2022 to January 2023. Specifically, colonies managed by beekeeping operation A had an average 21% reduction in population from September 2022 to January 2023, colonies managed by beekeeping operation B had an average of 36.2% population loss from August 2022 to January 2023, colonies managed by beekeeping operation C experienced a 44.1% average loss from September 2022 to January 2023, and colonies managed by beekeeping operation D had a 51.1% population loss from September 2022 to January 2023 (Figure 1, Supplementary Materials, Tables S1–S4) [72].

The number of colony deaths during the monitoring period varied by beekeeping operation. Specifically, 11 of the 61 (18%) monitored colonies (September to March) managed by beekeeping operation A died. Beekeeping operation B had 14 of 53 monitored colonies (26.4%) die from August to April. Only 1 of the 51 monitored colonies (2%) in beekeeping operation C died (September to January), and 5 of 31 monitored colonies (16.1%) managed by beekeeping operation D died (September to January) (Supplementary Materials, Tables S1–S4) [72]. We hypothesized that there may be previously uncharacterized viruses and/or virus strains (sequence variants) associated with colony population loss, colony death, and *V. destructor* infestation levels.

To investigate our hypotheses, colonies were categorized by beekeeping operation, sampling/assessment date, *V. destructor* mite infestation level, and change in colony population size (estimated as the change in the number of honey bee covered frames or ‘frame count’) over the sampling period [78]. The four population change groups were “high loss”, “healthy” (i.e., stable or population growth), “died during the monitoring period “, and “declined then recovered”, while the three *V. destructor* infestation categories were high, post-high, and low (Supplementary Materials, Table S5).

Of the 67 colonies represented in sequencing data, 19 were from beekeeping operation A, 18 were from beekeeping operation B, 14 were from beekeeping operation C, and 15 were from beekeeping operation D. Forty-three samples were obtained in August/September 2022, 52 in November 2022, and 46 in January 2023. Specifically, beekeeping operation A was located in South Central California; operation B was located near Chico, CA; operation C was located near Redding, CA, from August to November, and moved into almond orchards in the California central valley from January onward; and operation D was located near Yreka, CA, from August to November, and moved into almond orchards in the California central valley in January 2023.

At each sampling event, *V. destructor* mite infestation levels were assessed, and colony population size was estimated by frame count [78]. Eighty-nine samples were obtained from colonies with low *V. destructor* mite pressure. Thirty-six samples were obtained from colonies with high *V. destructor* mite pressure, and 16 were obtained from colonies with post-high *V. destructor* mite pressure. There were 75 samples from high loss colonies, 34 from samples from colonies with no population losses, 14 samples from colonies that died during the monitoring period, and 18 samples from colonies that recovered after initial population declines (Supplementary Materials, Table S5).

Ten honey bees from each colony-level sample were randomly selected and homogenized to yield lysates. Lysates from colonies matching the same criteria (e.g., beekeeping operation, sample date, colony health/population status, and *V. destructor* infestation level) were pooled (Supplementary Materials, Figure S5). This strategy yielded 67 separate pooled lysates, with a range of 1–7 colony-level lysates (representing 10–70 bees) withh amedian of 4 colony lysates (representing 40 bees). To maximize the number of viral sequencing reads, honey bee sample lysates were nuclease-treated prior to RNA extraction to degrade unencapsulated host RNA (mRNA, tRNA, rRNA, etc.), as described previously [4,61] (Figure 1, Supplementary Materials, Table S5).

### 3.2. RNA Sequencing (RNASeq) Libraries Representative of Honey Bee Sample Cohort

A total of 38 sequencing libraries were prepared from 141 honey bee samples, representing 67 unique colonies. Sequencing generated an average of 8,051,730 reads per library, which were assessed for quality, indexed, and trimmed for quality and to remove adaptor and index sequences (PRJNA1373327) (Supplementary Materials, Table S11).

**Figure 2:**
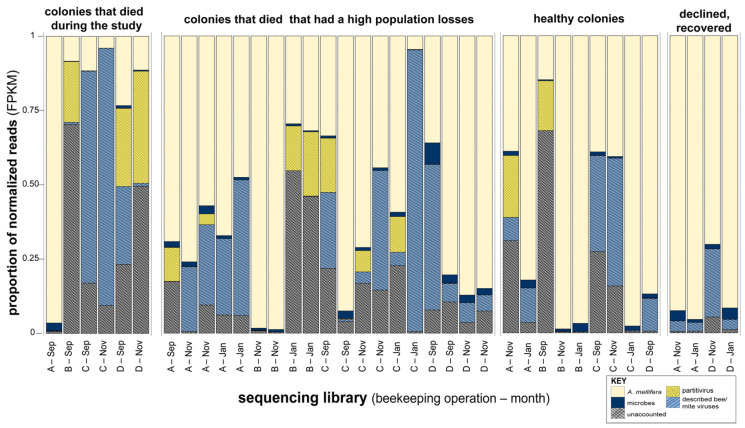
Composition of sequences in libraries characterized by honey bee colony population dynamics. Proportion of normalized reads from sequencing libraries categorized by health status: colonies that died during study, colonies that had high population losses, healthy colonies, and colonies that declined, then recovered. Sequencing libraries are labeled by beekeeping operation (A, B, C, and D) and month of sampling (Supplementary Materials, Tables S1–S4).

To enrich for viral reads, sequences were aligned to the honey bee genome (Amel_HAv3.1 NCBI RefSeq assembly GCF_003254395.2), with aligned reads being removed for virus discovery [84,85]. Additionally, reads were aligned to the Holobee Index (v2016.1), which contains honey bee associated microbe genomic sequences [86]. Reads aligning to these honey bee associated microbes were also removed (Figure 1). On average, 4,798,867 reads (59.74% of total reads) aligned to the honey bee genome, with a minimum of 142,441 and maximum of 9,983,204 (representing 1.83% to 94.37% of library reads, respectively). On average, 134,976 reads (1.56% of total reads) aligned to the nonviral Holobee index (v2016.1) with a minimum of 3,481 and maximum of 751,145 (representing 0.04% to 7.68% of library reads, respectively) (Figure 2, Supplementary Materials, Table S11).

De novo assembly of the remaining reads generated 152,898 contiguous sequences (contigs). A nonredundant list of 1825 contigs was generated by clustering similar contigs (greater than or equal to 95% nucleotide identity) and filtering any shorter than 1000 nt (Supplementary Materials, Files S1–S4). Putative viral contigs were identified by DIAMOND BLASTx alignment against NCBI’s virus RefSeq protein index (release 229) [91,92]. Open reading frames were predicted using Prodigal (v2.6.3), translated, and used as a query for HMMScan (v3.4) against the virus orthologous groups database (VOGDB, v227) [93,94,98]. Putative viral contigs were further refined by manual curation to remove sequences with false positive predicted homology to nonviral proteins (as determined by web-based BLASTn) and the removal of genome fragments (i.e., contigs that were appreciably shorter than the expected genome length). This resulted in a final, refined list of 64 contigs, including 16 characterized *A. mellifera* and/or *V. destructor* infecting viruses, eight characterized plant viruses (or genome segments), and 26 uncharacterized viruses or virus-like sequences (Supplementary Materials, Tables S12–S14). Abundance was assessed by aligning reads to these sequences, resulting in a range from 223 to 7,577,635 reads mapped (0.003% to 94.76% of total library reads), with an average of 1,961,543 reads aligning to the assembled putative virus sequences across sequencing libraries (23.57% of total library reads). Putative plant viruses comprised a small fraction of reads (~0.02% of total sequencing reads) (Supplementary Materials, Table S13). To make comparisons of viral abundance across sequencing libraries, read counts were normalized to fragments per kilobase of transcript per million mapped reads (FPKM) (Supplementary Materials, Table S15). This method takes the total number of reads per sequencing library and the length of the transcript (or, in this case, putative viral genome) into account. Reads were aligned to the *A. mellifera* genome (Amel_Hav3.1), honey bee associated microbes (HoloBee index), plant viruses, partitivirus-like sequences, and known bee viruses; unaligned reads were classified as “unaccounted”. A greater proportion of reads aligned to viruses in libraries representing colonies that died or experienced high population loss during the monitoring period relative to those representing colonies with stable or growing populations or from those with populations that declined and recovered (Figure 2).

### 3.3. Previously Characterized Virus Sequences Were Genetically Similar

Of the 64 contigs identified herein, 23 nonredundant, genome-length contigs were similar to 16 well-characterized bee and/or *V. destructor* mite viruses (including those with multipartite genomes). These included acute bee paralysis virus (ABPV), Apis rhabdovirus 1 (ARV-1), black queen cell virus (BQCV), chronic bee paralysis virus (CBPV), deformed wing virus A and B (DWV-A, DWV-B), Lake Sinai viruses 1–4 (LSV-1, LSV-2, LSV-3, LSV-4), sacbrood virus (SBV), tobacco ringspot virus (TRSV), Varroa tymo-like virus, Varroa destructor virus 3 (VDV-3), Varroa orthomyxovirus 1 (VOV-1), and Varroa destructor virus 9 (VDV-9).

To assess the similarity of viruses across beekeeping operations (representing a broad geographic range), assembled virus sequences from each library were aligned to one another. The sequences of common honey bee infecting viruses including ARV-1, ABPV, BQCV, DWV-A, DWV-B, and SBV were similar across beekeeping operations, with greater than 97% nucleotide identity (Supplementary Materials, Table S8). As colonies were located more than 80 km from each other, and the flight range of honey bees is typically ~5 km, colonies were geographically isolated from one another, indicating the sequences reported herein represent the dominant circulating strains.

Most of the previously described honey bee and/or *V. destructor* infecting viruses sequenced herein were similar to published sequences (Table 1) (NCBI accessed September 2025) [99]. Nucleotide differences between the assembled sequences and previously reported DWV-A and DWV-B genomes were evenly distributed, suggesting that the sequences identified herein describe the consensus quasispecies sequences and are not recombinants (Table 1). The Lake Sinai virus group includes highly divergent members, including 22 variants of LSV-1 and 44 variants of LSV-2 sequences deposited on NCBI (accessed September 2025) [80,99,100]. Three unique LSV-2 sequence variants (i.e., LSV-2-CA-1, LSV-2-CA-2, and LSV-2-CA-3) (PX726312, PX726313, PX726314) and two unique LSV-3 sequence variants (LSV-3-CA-1 and LSV-3-CA-2) (PX726315, PX726316) were identified in this study (Table 1).

The LSV-2 variants reported herein shared 89.3% to 93.7% nucleotide identity to one another. These nucleotide differences were evenly distributed across the three sequences. The LSV-3 variants were more distinct (i.e., 84.4% nt identity). While unique and distinct from each other, these sequence variants were similar to previously reported LSV strains (Table 1). The LSV-2 variants shared 91.3% to 96.1% identity to previously reported LSV-2 strains, and the LSV-3 variants shared 96.1% and 98.8% identity to reported LSV-3 strains (Supplementary Materials, Table S16) [99,101,102]. Nucleotide variation was evenly distributed across sequences, supporting that these consensus sequences represent distinct variants. In addition, we ensured the specificity of mapped reads by aligning using either single- or multi-mapping modes. This strategy revealed that the number of reads assigned to each contig was similar whether single- or multi-mapping modes were utilized, indicating that individual reads were not aligning to multiple LSV-2 or LSV-3 sequences (Supplementary Materials, Table S10).

### 3.4. New Lake Sinai Virus and Acute Bee Paralysis Virus Variants Discovered

Two new virus sequence variants, acute bee paralysis virus CA-2022 (ABPV-CA-22, PX726311) and Lake Sinai virus-9 (LSV-9, PX726310), were identified in beekeeping operation C, which experienced high population declines (i.e., 44%) over the course of this study (Supplementary Materials, File S1). Specifically, ABPV-CA-2022 was abundant in sequencing library 24, which represented seven colonies in beekeeping operation C sampled in November 2022. This ABPV variant was 90.8% identical to ABPV isolate No11_Am029-XJ2018 (GenBank MZ821781), with nucleotide differences evenly distributed across the 9660 nt genome (Supplementary Materials, Figure S1). A total of 3883 FPKM in this library was assigned to ABPV-CA-2022 (representing a coverage of 5168×). Albeit in lower abundance (201 FPKM), this ABPV variant was detected in sequence libraries 30 and 32, representing nine colonies sampled in August 2022 from beekeeping operation D, which experienced 51% bee population loss during the monitoring period. Interestingly, ABPV-CA-2022 was abundant (4104 FPKM) in a library generated from eight colonies sampled in 2022 from a Montana-based beekeeping operation that experienced high colony losses. Detection across beekeeping operations and states suggests this variant is geographically widespread.

A new member of the Lake Sinai virus group, LSV-9, was the most abundant virus (90,093 FPKM) in library 21 (54.2% of total reads). This library represented three colonies in beekeeping operation C sampled in September 2022. This operation experienced high average population decline (44%) over the course of the study. However, LSV-9 was not detected in libraries made from the same colonies sampled at later dates (November 2022 and January 2023; libraries 25 and 28). LSV-9 was 83.7% identical to LSV-8 Goryeong (GenBank OR496495) at the nucleotide level, and the variation was evenly distributed across the 6,014 nt genome (Supplementary Materials, Figure S2). The LSV-9 RdRp, capsid, and nonstructural polyprotein amino acid sequences shared 90.4%, 95.4%, and 87.1% amino acid identity, respectively, with LSV-8 Goryeong.

### 3.5. Six Abundant Putative New Virus Sequences Were Identified, Including Three New Widespread Partitivirus-like Sequences

The sample processing, RNA sequencing, and bioinformatic analyses utilized in this study facilitated identification of new putative virus sequences. After de novo assembly using SPAdes, putative viral contigs were identified by BLASTx and HMMscan against the Refseq virus protein database (release 231) and VOGDB (v227), respectively [91,92,93,103]. After manual curation to remove false positives and genome fragments, approximately 26 contigs representing putative new viruses or virus-like sequences were identified (Supplementary Materials, Table S14). These sequences shared less than 70% nucleotide identity with known viruses. Only six of these putative new virus sequences had more than 1000 reads in a single sequencing library. Of the three less abundant sequences, one is a putative plant virus with homology to a virus in the Solemoviridae family, sharing 54.3% nt identity to soybean yellow common mosaic virus (LC332542). Due to detection in a Southern California-based beekeeping operation, this putative virus was named “Southern California sobemovirus 1” (PX726319). The second is a putative plant virus with homology to a virus in the Potexviridae family, sharing 65.1% nt identity to Alstromeria virus X (NC_007408) [104]. As this sequence was detected in two Northern California based beekeeping operations, this putative virus was named “Northern California potexvirus 1” (PX726319). The third was similar to an unclassified virus identified in mosquitoes (58.7% nt identity) and a dicistrovirus identified in aphids, Aphis glycines virus 3 (RdRp 30.3% aa identity) [105,106]. As this sequence was detected in a Southern California based beekeeping operation, this putative virus was named “Southern California dicistrovirus 1” (PX726319). The abundance of these sequences was relatively low; combined reads represented 0.01% of total reads, and abundance was only 691 FPKM (Supplementary Materials, Table S14).

**Figure 3:**
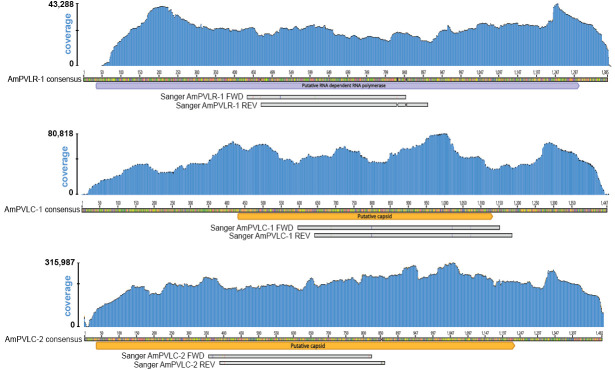
New partitivirus-like sequences identified in honey bees. Schematics of the three partitivirus-like sequences identified in honey bee samples obtained from California based beekeeping operations; read depth illustrated by blue shaded region above each sequence. The average coverage of Apis mellifera partitivirus-like RdRp 1 (AmPVLR-1), Apis mellifera partitivirus-like capsid 1 (AmPVLC-1), and Apis mellifera partitivirus-like capsid 2 (AmPVLC-2) across sequencing libraries was 50,963×, 80,363×, and 87,088×, respectively. Open-reading-frames-encoding-predicted proteins (based on homology) are annotated below the sequences, with AmPVLR-1 encoding a putative RNA-dependent RNA polymerase (RdRp), and AmPVLC-1 and AmPVLC-2 encoding putative capsid proteins. Sanger sequencing was used to validate the assemblies. Sequenced regions (gray bars) shared >99% nucleotide identity with the assembled sequences (colored lines indicate nucleotide mismatches).

In contrast, three new partitivirus-like sequences were prevalent and abundant. These sequences were named Apis mellifera-associated partitivirus-like capsid 1 (AmPVLC-1, PX726307), Apis mellifera-associated partitivirus-like capsid 2 (AmPVLC-2, PX726308), and Apis mellifera-associated partitivirus-like RdRp 1 (AmPVLR-1, PX726309) (Figure 3, Supplementary Materials, File S1). The AmPVLC-1 sequence is 1399 nt long and encodes an open reading frame (ORF) that is 234 amino acids (aa) and most similar to Tarsiger cyanurus CRESS-DNA-virus coat protein (UniProt A0A4Y5QKV2) (e-value 2.8 × 10^−8^). The AmPVLC-2 sequence is 1472 nt long and encodes an ORF of 399 aa that is most similar to Osugoroshi uncharacterized protein (UniProt A0A7R7T203) (e-value 2.5 × 10^−11^) [107]. The AmPVLR-1 sequence is 1398 nt long and encodes an ORF of 425 aa, which is homologous to the Ripivanb virus RdRp (GenBank WZH59083.1) (51.33% aa identity, BLASTx e-value = 2.0 × 10^−147^) (Supplementary Materials, Table S17) [108]. Sanger sequencing of an ~500 nt region of each of these sequences verified the assembled sequences (>99% nucleotide identity) (Figure 3). These sequences had very high coverage in positive sequencing libraries (Table 1, Supplementary Materials, Table S15). These sequences were most similar to various partitiviruses, which are viruses comprised of multipartite dsRNA genomes capable of infecting animals, plants, and fungi [109]. Since AmPVLC-1, AmPVLC-2, and AmPVLR-1 sequences did not always co-occur, they are not likely components of a multipartite partitivirus genome.

### 3.6. Hubei Partiti-like Virus 34 and Apis mellifera Associated Partiti-like Virus 1 Were Present and Abundant Across All Beekeeping Operations

In addition, two previously described partitivirus-like segments, Apis mellifera partiti-like virus 1 (AmPLV1) and Hubei partiti-like virus 34 (HPLV-34), previously detected in honey bee samples, were prevalent and abundant in the sample cohorts of this study [69,70,102,110]. The genome segments are 1456 nt and 1475 nt long, respectively, and each contains one major ORF encoding a putative RdRp (Supplementary Materials, Figure S3). These two previously described partitivirus sequences and the three partitiviruses identified in this study (Apis mellifera partitivirus-like capsid 1 (AmPVLC-1), Apis mellifera partitivirus-like capsid 2 (AmPVLC-2), and Apis mellifera partitivirus-like RNA-dependent RNA polymerase 1 (AmPVLR-1)) were detected in colony-level samples obtained from all four California-based beekeeping operations at all sample dates, as well as in samples from colonies in Washington and Montana, suggesting a widespread distribution. Seventeen sequencing libraries (44.7% of total libraries) were positive for at least one of these partitivirus-like sequences. Partitivirus sequences were highly abundant in these positive libraries, with an average total partitivirus sequence abundance of 1,771,678 FPKM (Table 1, Supplementary Materials, Table S15). Partitivirus-like sequences comprised 27% of total viral FPKM across all sequencing libraries (30,129,850 of 111,535,868 total viral FPKM).

### 3.7. Correlation of Partitivirus-like Sequences

Since partitiviruses are multi-segmented dsRNA viruses, we examined potential correlations between AmPVLC-1, AmPVLC-2, AmPVLR-1, AmPLV1, and HPLV-34 to assess whether these sequences comprise a multipartite viral genome. The abundance (FPKM) of AmPVLC-1 and AmPVLC-2 (R^2^ = 0.99, *p* = 2 × 10^−16^), HPLV-34 and AmPVLC-2 (R^2^ = 0.89, *p* = 2 × 10^−16^), HPLV-34 and AmPVLC-1 (R^2^ = 0.89, *p* = 2 × 10^−16^), and AmPVLC-1, AmPVLC-2, and HPLV-34 (R^2^ = 0.99, *p* = 2 × 10^−16^) were positively correlated across sequencing libraries (Spearman’s correlation) (Supplementary Materials, Figure S4). In addition, partitivirus presence at the colony level (n = 10 bees) was examined using PCR. Colonies that comprised libraries that had high partitivirus abundance (>500,000 reads aligned) were screened. Specifically, 46% (19/41) of colonies were positive for AmPVLC-2, 37% (15/41) were positive for AmPVLC-1, 41% (17/41) were positive for AmPVLR-1, 22% were positive for AmPLV1 (9/41), and 90% were positive for HPLV-34 (37/41) (Supplementary Materials, Figure S5, Supplementary Materials, Table S18). The presence of AmPVLC-2, AmPVLC-1, and HPLV-34 was correlated at the colony level, with readily detectable PCR products for all three sequences in 15 of the 41 colony-level samples examined (Supplementary Materials, Figure S5).

To further support these observations, quantitative PCR (qPCR) was performed on a representative subset of colony-level samples to assess AmPVLC-1, AmPVLC-2, and HPLV-34 abundance. Quantitative analyses revealed correlations between AmPVLC-1, AmPVLC-2, and HPLV-34 (R^2^ = 0.97, *p* < 2.2 × 10^−16^ for the correlation between AmPVLC-1 and AmPVLC-2, R^2^ = 0.93, *p* < 2.2 × 10^−16^ for the correlation between AmPVLC1 and HPLV-34, R^2^ = 0.95, *p* < 2.2 × 10^−16^ for the correlation between AmPVLC-2 and HPLV-34, and R^2^ = 0.97 for the correlation between AmPVLC-1, AmPVLC-2, and HPLV-34) (Supplementary Materials, Figure S6). These results suggest that AmPVLC-2, AmPVLC-1, and HPLV-34 comprise three segments of a single partitivirus genome. However, additional analyses are required to validate this hypothesis.

In addition, two unclassified contigs correlated with partitivirus sequence abundance. The two unclassified contigs (i.e., highly abundant unknown contig 1, PX72632 and highly abundant unknown contig 2, PX726323) were prevalent and abundant in samples from all four beekeeping operations at all sample dates; they accounted for 4.38% of all sequencing reads. While their ~1.4 kb length and predicted ORFs suggest they may represent partiti-like virus sequences, homology-based searches yielded no significant results. Unknown highly abundant contig 1 was correlated with AmPVLC-1, AmPVLC-2, and HPLV-34 (R^2^ = 0.81, 0.56, and 0.17, *p* = 2 × 10^−16^, 7.6 × 10^−9^, and 0.0063, respectively, Spearman’s correlation). Unknown highly abundant contig 2 was correlated with AmPVLR-1 and AmPLV1 (R^2^ = 0.92 and 0.50, *p* = ~2 × 10^−16^ and 1.2 × 10^−7^, respectively, Spearman’s correlation) (Supplementary Materials, Figure S7). It is possible these contigs are divergent partitivirus-like sequences encoding unknown protein products. Their correlation with the other identified partiti-virus like sequences suggests they may comprise additional segments of the same partitivirus genome.

### 3.8. Sequencing Libraries Representing Colonies That Died During the Monitoring Period Harbored Greater Virus Abundance

**Figure 4:**
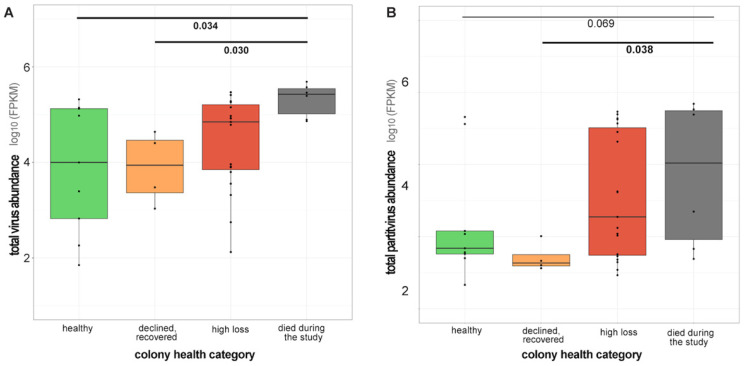
Virus abundance was greater in libraries representing colonies that died during the study. (**A**) Total virus abundance was greater in libraries representing colonies that died during the sampling period relative to healthy colonies, which had populations that were stable or increased (*p* = 0.034) or those that experienced initial population declines, but subsequently recovered (declined/recovered) (*p* = 0.030). Libraries generated from healthy colonies had an average of 59,544 viral FPKM, libraries generated from colonies that declined and recovered had an average abundance of 9957 viral FPKM, whereas libraries representing colonies that suffered high population losses had an average virus abundance of 95,892 FPKM and libraries representing colonies that died during the study had an average of 245,862 viral FPKM. (**B**) Total partitivirus-like sequence abundance was greater in libraries representing colonies that died during the sampling period relative to those that experienced initial population declines but subsequently recovered (declined/recovered) (*p* = 0.038). Libraries generated from colonies that died during the study had an average partitivirus-like abundance of 178,522 FPKM, whereas libraries generated from colonies that experienced population declines but recovered had very low levels of partitivirus-like sequences (average 38 FPKM). Libraries generated from healthy colonies had an average of 38,061 FPKM partitivirus-like sequence abundance and libraries generated from colonies that suffered high population declines had an average virus abundance of 62,043 FPKM.

Viral reads (representing previously characterized viruses, undescribed viruses, and partitivirus-like sequences) comprised a greater proportion of reads in libraries representing colonies that died during the monitoring period or experienced population declines. Specifically, 44% of reads were viral in sequencing libraries representing colonies that died during the monitoring period (n = 6) and 24% of reads were viral in libraries representing colonies that experienced high population losses (n = 19). Of the libraries representing colonies that died during the monitoring period, average viral abundance was 245,862 FPKM. Average virus abundance was 95,892 FPKM in libraries representing colonies that suffered high population losses (Figure 2 and Figure 4A). Conversely, the proportion of viral reads in libraries representing colonies with stable populations (16% of reads, n = 9 libraries) or colonies that experienced population declines and then recovery (8% of reads, n = 4 libraries) was lower. Similarly, libraries representing colonies harbored lower average virus abundance (59,544 FPKM). Libraries generated from samples from colonies that experienced population declines that subsequently recovered had an average viral abundance of 9957 FPKM. Virus abundance was greater in libraries representing colonies that died during the monitoring period than in colonies maintaining stable populations (*p* = 0.034) or colonies that recovered after population declines (*p* = 0.030, Dunn’s Test with Bonferroni correction) (Figure 4A).

**Figure 5:**
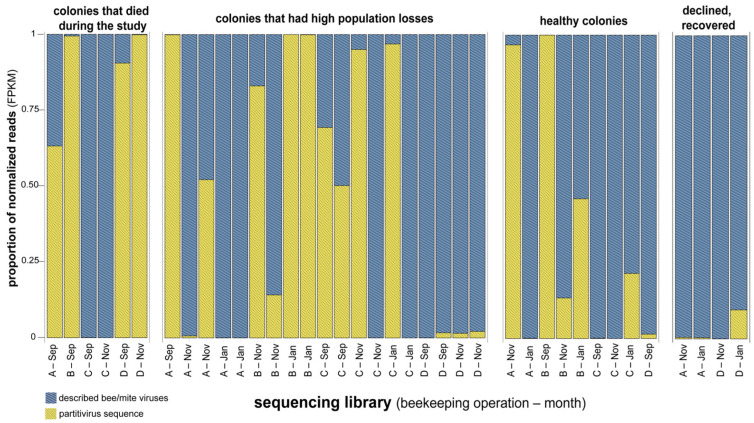
Overview of virus sequence composition in libraries characterized by population dynamics. The proportion of partitivirus-like sequences (yellow dotted) compared to the proportion of reads that align to all previously described bee and/or mite viruses (blue grid). Sequencing libraries are labeled by beekeeping operation (A, B, C, and D) and sample month (Supplementary Materials, Tables S1–S4). Most sequencing libraries had high proportions of partivirus-like sequences, including libraries representing colonies that died during the study (69.3%), libraries representing colonies that had high population losses (67.1%), and libraries generated from healthy colonies (58.0%). Whereas, libraries representing colonies that experienced population decline and then recovered harbored a low proportion of partitivirus-like sequences (0.2% of total viral FPKM).

Partitivirus-like sequences comprised the majority of viral reads in libraries representing colonies that died during the monitoring period or experienced high population losses (69% and 67% of total viral FPKM, respectively). These proportions were greater than those from libraries with stable populations (58% of FPKM) and those that recovered after population decline (<1% of viral FPKM) (Figure 4B, Figure 5). Libraries representing colonies that died during the monitoring period had greater partitivirus-like sequence abundance relative to libraries representing colonies that experienced initial population declines and then recovered (*p* = 0.038, Dunn’s Test with Bonferroni correction). Partitivirus-like sequence abundance was greater, but not significantly so, in libraries representing colonies that died during the monitoring period relative to those representing colonies that did not experience population decline (*p* = 0.069, Dunn’s Test with Bonferroni correction) (Figure 4B and Figure 5).

### 3.9. Libraries Representing Colonies with High Varroa destructor Mite Infestation Had Higher DWV Levels

**Figure 6:**
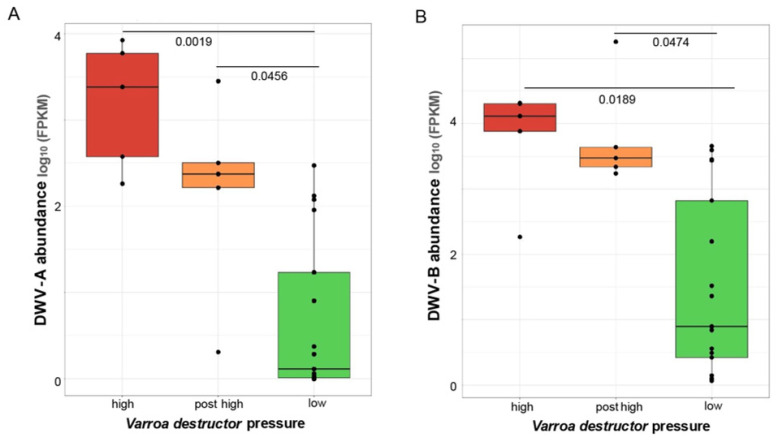
Higher DWV abundance associated with *Varroa destructor* mite infestation. DWV-A and DWV-B abundance is greater in libraries generated from samples from colonies with high *V. destructor* pressure and samples obtained after mite treatment (post-high), relative to libraries generated from samples from colonies with low *V. destructor* mite level. (**A**) Average DWV-A abundance across libraries representing high *V. destructor*, post-high *V. destructor*, and low *V. destructor* samples was 3460 FPKM, 770 FPKM, and 39 FPKM, respectively. (**B**) Average DWV-B abundance was greatest in libraries representing samples from colonies after *V. destructor* mite pressure (38,147 FPKM), followed by libraries from colonies with high *V. destructor* mite pressure (12,324 FPKM) and libraries representing samples from colonies with low mite pressure (879 FPKM).

Libraries representing samples from honey bee colonies with high levels of *V.* *destruct* mites (n = 5), or libraries from samples obtained from colonies after high mite levels (post-high mite) (n = 5) had an average of 29.43% and 34.6% virus reads, respectively. These percentages were greater than those observed from libraries representing colonies that had low mite levels (n = 12) (20.0%). The abundance of DWV-A (FPKM) was significantly greater in libraries representing high mite and post-high mite samples relative to libraries representing low mite samples (*p* = 0.0019 and 0.0456, Dunn’s Test with Bonferroni correction) (Figure 6). Similarly, DWV-B abundance (FPKM) was greater in libraries representing high mite and post-high mite samples relative to libraries representing low mite samples (Dunn’s Test, *p* = 0.0189 and *p* = 0.0474, Dunn’s Test with Bonferroni correction) (Figure 6). Importantly, most DWV-B abundance was contributed by a single sequencing library from post-high-mite samples (sequencing library 29).

### 3.10. Differences In Virus Abundance and Composition by Assessment Timepoint

Average DWV-B abundance was greater in January 2023 (17,424 FPKM) relative to August/September 2022 (4,485 FPKM) and November (4,071 FPKM). The LSV-2 variants, LSV-9, AmPLV1, and AmPVLR-1, were more abundant at earlier sampling events (August/September and November 2022) relative to January 2023. Except for LSV-2-CA-3, these viruses were virtually undetected in January, despite relatively stable abundance from August/September to November. Finally, AmPLV1 and AmPVLR-1 abundances (FPKM) were greater in August/September relative to January (Dunn’s test, *p* = 0.0186 and 0.0058, respectively) (Supplementary Materials, Figures S8–S10). These findings are in line with prior California based colony monitoring surveys that found virus abundance varied by season and that DWV abundance peaked in fall [79]. Since partitivirus-like sequence abudance correlated with colony death, apparent seasonality may reflect that colonies that died before January were not sampled that month. In addition, thetrend in abundance across the sampling timepoint was not reflected in each individual library. For example, LSV-9 was only detected in a single library representing colonies sampled in August. Thus, conclusions based on average data may be inaccurate.

### 3.11. Individual Sequencing Libraries Are Not Equally Represented by Average Group Virus Composition and Abundance

**Figure 7:**
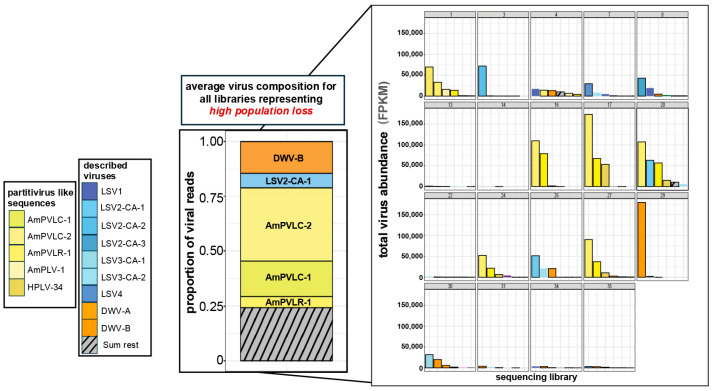
Individual sequencing libraries are not equally represented by average categorical virus composition and abundance. The five most abundant viruses for each sequencing library representing honey bee colonies that experienced high population losses are graphed individually (n = 19), with partitivirus-like sequences in yellow shades and unassigned reads in gray (small bar charts). The average virus composition across all these libraries illustrates high relative abundance of DWV-B, LSV-2-CA2, and partitivirus-like sequences (large bar chart). Comparison of the top five most abundant viruses in the graph representing the average reveals that not all individual sequencing libraries are equally represented.

The average virus composition and abundance for a given category (i.e., high population loss) is not representative of all individual sequencing libraries. For example, the average virus abundance in libraries representing honey bee colonies with high population losses was 91,528 FPKM, and the five most abundant virus sequences were DWV-B, LSV-2-CA-1, AmPVLC-2, AmPVLC-1, and AmPVLR-1. However, six of the nineteen (31.6%) libraries in this category had low virus levels (<10,000 viral FPKM), and only a few libraries contributed most of the virus sequences (Figure 7). For example, most of the DWV-B abundance was contributed by a single library (i.e., library 29). In some cases, a single library contributed nearly all reads for a given virus (i.e., library 21 contributing 99% of LSV-9 abundance). Thus, even among colonies with the same underlying characteristics, such as management by the same beekeeping operation, similar increases and/or decreases in population, and *V. destructor* pressure, virus composition and abundance varied. Of the 38 libraries, 15 harbored low virus abundance (<10,000 viral FPKM total). The five libraries with highest virus abundance contributed ~42.9% of total virus abundance. Thus, group averages were dominated by reads from a few highly abundant libraries.

## 4. Discussion

Partitivirus-like sequences, which were detected in honey bee samples from four California based migratory beekeeping operations in 2022–2023, were highly abundant (i.e., present in 44% of sequencing libraries). Three of these sequences were previously undescribed (AmPVLC-1, AmPVLC-2, and AmPVLR-1), while two had been described and detected in honey bees (HPLV-34 and AmPLV1) (61, 87–89). Interestingly, AmPVLC-2 (the most abundant virus-like sequence in this study) was most similar to Osugoroshi virus, a virus described in the Oriental tea totrix moth (*Homona magnanima*) that correlated with the male-killing phenotype in this species [107]. However, homology was low (3% query coverage with 84% nt identity). Partitiviruses replicate in numerous insects and negatively impact the fitness of *D. biauria*, *Spodoptera exempta,* and *H. magnamina*. In addition, partitiviruses have also been associated with male-killing in *Drosophila* [111].

While it has not yet been shown that they replicate in honey bees, high abundance across libraries suggests these sequences represent segments of one or more partitiviruses that are capable of replication in honey bees. Specifically, significant correlations between AmPVLC-1, AmPVLC-2, and HPLV-34 indicate these three sequences may comprise segments of the same multipartite partitivirus genome, with HPLV-34 encoding the RdRp, and AmPVLC-1 and AmPVLC-2 encoding two putative capsid proteins. The detection of partitivirus-like sequences across beekeeping operations spanning multiple states (California, Washington, Montana) suggests their wide geographic range.

Partitivirus-like sequences made up a greater proportion of total viral reads (FPKM) in libraries representing colonies that died during the monitoring period and colonies that experienced high population losses, relative to libraries representing colonies not experiencing population loss. Similarly, total partitivirus-like sequence abundance was greater in libraries representing colonies that died during the monitoring period relative to those representing colonies that survived. Due to the observational nature of this study, the associations between virus abundance and colony health do not necessarily indicate that viruses are responsible for colony population size or mortality. Further studies are required to evaluate the potential impact of these viruses on honey bee colony health. While the impact of partitiviruses on bees is unknown and no overt morphological symptoms have been associated with these infections, viruses are obligate intracellular pathogens that utilize host cellular resources to replicate, therefore they may be energetically costly for the host—particularly if immune responses are mounted. Honey bees often harbor high virus loads in the absence of overt morphological symptoms [5,37,77,79,112]. Inapparent DWV infections in honey bees have been shown to negatively impact flight performance [113,114]. At the colony level, virus prevalence and abundance has also been correlated with colony losses [28,37,38,46,79,115,116,117,118]. Thus, it is possible that viruses, including partitiviruses, negatively impact bee hosts at the individual bee and/or colony level even in the absence of symptomatic infection, though additional studies are required to evaluate the potential impact of partitiviruses on honey bee health.

In addition to these three partitivirus-like sequences, a new member of the Lake Sinai virus group (LSV-9) and a sequence variant of ABPV (ABPV-CA-22) were discovered. These viruses were detected in operations experiencing high losses; therefore, it is possible that they contributed to colony deaths. However, due to the observational nature of this study, additional studies are required to elucidate the potential impact of specific viruses and virus strains on honey bee health. The association of ABPV with high colony losses in 2025 makes the discovery of this ABPV variant particularly interesting, as this sequence variant was detected in California and Montana [117].

Twenty-two other putative new virus sequences or sequence fragments were identified, although most had relatively low abundance (accounting for <0.00015% of sequencing reads). There were three new putative viral sequences with >1000 reads in a single sequencing library. These included two putative new plant viruses sharing homology to the families Solemoviridae and Potexviridae and one putative new insect virus sharing homology to Dicistroviridae. While some plant viruses, such as Tobacco ringspot virus (TRSV), are capable of replication in honey bees, it is possible that thereads detected in our study were contributed from virus-contaminated pollen [59,119,120]. The detection of Potexviridae across multiple beekeeping operations suggests this virus may have broad geographic distribution.

Sequences representing common viruses assembled from reads representing geographically isolated beekeeping operations were similar; most had over 97% nucleotide identity. Nucleotide similarity of greater than 90% is attributable to RNA diversity generated during genome replication [121]. Thus, the high degree of nucleotide identity suggests these viruses are the dominant virus strains currently circulating in California.

Since sequencing libraries were grouped by colony-level characteristics, the average virus composition and abundance for a given group (i.e., high loss colonies) was not representative of all individual libraries included in that category. Thus, the results from this study and future sequencing-based studies focused on virome characterization should be interpreted with caution, since conclusions drawn from averages may not be representative of individual libraries; associations between virus presence and abundance and colony population size and/or health status do not necessarily indicate causal relationships.

Libraries generated from honey bee samples obtained from colonies with high *V. destructor* mite pressure and samples obtained from colonies after they experienced high mite pressure had greater DWV-A and DWV-B abundance relative to libraries generated from samples obtained from colonies with low mite levels. *V. destructor* mites are replication-competent vectors of DWV [54,122], and our results underscore the importance of mite-mediated DWV transmission and reveal that virus levels remain high after mite infestation is controlled.

## 5. Conclusions

Here, we report the discovery of three new partitivirus-like sequences that are highly abundant across four California based beekeeping operations, as well as one Washington and one Montana based beekeeping operation. Additionally, we detected two abundant previously described partitivirus-like sequences (HPLV-34 and AmPLV1) and 16 well-characterized bee and/or *V. destructor* infecting viruses, a new Lake Sinai virus (LSV-9), and a sequence variant of ABPV (ABPV-CA-22). Overall, total virus abundance and partitivirus-like sequence abundance was greater in libraries representing colonies that died during the monitoring period. These results further support that viruses are associated with honey bee colony deaths and indicate that further investigations on the effects of viruses on individual bee and colony health are warranted.

## Figures and Tables

**Table 1:** Overview of previously described bee- and/or mite-associated viruses sequenced in this study.

Virus	Length (nt)	% nt Identity to Most Similar Virus	Sequencing Libraries Positive	Total Reads	Total FPKM	Average Coverage
acute bee paralysis virus	9660	90.8	3/38	348,783	4084	5415
Apis mellifera associated partiti-like virus 1	1456	96.6	7/38	1,982,504	165,643	204,241
Apis rhabdovirus 1	14,589	99.7	10/38	696,441	6057	7162
black queen cell virus	8526	96.4	8/38	545,081	7640	9590
chronic bee paralysis virus RNA1	3602	96.7	0/38	425	16	18
chronic bee paralysis virus RNA2	2239	92.3	0/38	266	16	18
deformed wing virus—A	10,221	99.5	16/38	1,886,851	22,267	27,691
deformed wing virus—B	10,264	99.7	25/38	24,939,879	306,562	364,476
Hubei partiti-like virus 34	1475	99.6	13/38	1,106,510	93,910	112,526
Lake Sinai virus 1	6048	98.2	11/38	2,331,743	49,291	57,831
Lake Sinai virus 2—CA-1	5953	91.3	9/38	19,889,636	403,108	501,167
Lake Sinai virus 2—CA_2	5994	94.6	9/38	4,098,226	88,706	102,558
Lake Sinai virus 2—CA-3	6044	94.3	8/38	14,464,143	261,105	358,971
Lake Sinai virus 3—CA-1	6128	96.1	7/38	2,313,434	45,341	56,628
Lake Sinai virus 3—CA-2	6195	98.8	10/38	2,474,919	45,554	59,925
Lake Sinai virus 4	6122	95.9	9/38	1,838,938	35,492	45,057
Lake Sinai virus 9	6013	83.7	1/38	3,900,005	90,096	97,289
sacbrood virus	8831	99.5	6/38	367,862	5379	6248
Tobacco ringspot virus RNA1	7145	96.0	3/38	15,952	270	335
Tobacco ringspot virus RNA2	3565	95.1	1/38	9110	308	383
Varroa destructor virus 3	3994	96.8	0/38	306	10	11
Varroa destructor virus 9	1512	90.0	0/38	475	13	47
Varroa orthomyxovirus1—glycoprotein	1679	99.3	1/38	12,690	924	1134
Varroa orthomyxovirus1—NP	1495	99.2	1/38	5126	419	514
Varroa orthomyxovirus1—PB2	2342	99.1	1/38	4917	257	315
Varroa tymo-like virus	6186	94.2	1/38	3062	57	74

## Data Availability

The majority of the data generated or analyzed during this study are included in this published article and its Supplementary Materials, files (available online). Additional data are available from the corresponding author upon request, and sequence data are available on GenBank and the NCBI Sequence Read Archive (BioProject ID PRJNA1373327).

## Supplementary Materials

The following supporting information can be downloaded at: https://www.mdpi.com/article/10.3390/v18030334/s1, Excel file with sheets containing Tables S1–S18, Combined PDF document containing Supplementary Materials, Figures S1–S10, Supplementary Materials, Text S1. Supplementary Materials, data including raw qPCR data files, raw outputs of HMMscan, and raw outputs of DIAMOND searches are available in the “Supplementary Materials, Data” folder. Supplementary Materials, Figure titles are listed below for review: Table S1: Colony level data/characteristics for beekeeping operation A; Table S2: Colony level data/characteristics for beekeeping operation B; Table S3: Colony level data/characteristics for beekeeping operation C; Table S4: Colony level data/characteristics for beekeeping operation D; Table S5: Summary of pooling strategy (i.e., colonies included in each sequencing library) and individual colony-level characteristics for each pooled sequencing library; Table S6: Primers used in this study; Table S7: Raw qPCR data and standard curves generated for quantification of partitivirus-like sequence abundance; Table S8: Similarity of commonly detected bee viruses across sequencing libraries; Table S9: Comparison of mapping stringency settings in HISAT2; Table S10: Number of reads mapped using either single- or multi-mapping enabled (HISAT2) to LSV-2/LSV-3 variants, and DWV-A/DWV-B; Table S11: Overview of read composition per sequencing library; Table S12: Previously described well-characterized bee and/or *V. destructor* mite viruses sequenced in this study; Table S13: Plant viruses sequenced in this study; Table S14: Undescribed virus-like sequences sequenced in this study; Table S15: Normalized read count (FPKM) for each virus per sequencing library; Table S16: Similarity of the LSV-2 and LSV-3 variants sequenced in this study; Table S17: Characterization of three undescribed partitivirus-like sequences sequenced in this study; Table S18: Proportion of individual colonies that were positive for each partitivirus-like sequence; Figure S1: Acute bee paralysis (ABPV-CA-22) variant; Figure S2: Lake Sinai virus 9; Figure S3: Partitivirus-like genome schematics; Figure S4: AmPVLC-1, AmPVLC-2, and AmPVLR-1 abundance was positively correlated in sequence libraries; Figure S5: Co-occurrence of AmPVLC-1, AmPVLC-2, and AmPVLR-1 at the colony level; Figure S6: Abundance of AmPVLC-1, AmPVLC-2, and HPLV-34 correlates at the colony level; Figure S7: Two unclassified contigs correlated with partitivirus sequence abundance; Figure S8: Virus abundance in sequencing libraries from August and September 2022; Figure S9: Virus abundance in sequencing libraries November 2022; Figure S10: Virus abundance in sequencing libraries from January 2023; Text S1: Detailed materials and methods.
