# Supplementary material for: Honey Bee Viromes from Beekeeping Operations Experiencing High Losses in 2022–2023
Source: Viruses. 2026 Mar 9;18(3):334. doi: 10.3390/v18030334 (PMC13030351; doi:10.3390/v18030334)
Supplement: Supplementary file 1 [file viruses-18-00334-s001.zip › Supplementary Material Text S1.pdf]

Detailed Materials and methods

2.1. Honey bee samples

Honey bee samples were obtained from four different commercial beekeeping operations in California. Individual colonies were repeatedly assessed and sampled from all operations in either August or September 2022, November 2022, and January 2023. Beekeeping operations A and B were additionally sampled in March and April 2023, respectively (Supplementary Tables S1-S4). Honey bee samples were stored at –80°C until analyses.

At each sampling event, *Varroa destructor* mite infestation levels were assessed and colony population size, which is a proxy for colony health, was estimated by count of honey bee covered frames [1–3]. Four categories were utilized to describe honey bee colony population dynamics. Specifically, colonies with populations that decreased by more than 40% over the monitoring period were considered "high loss" colonies. Colonies that either maintained their initial population or increased in population over the course of the study were considered "healthy" colonies. Colonies with populations that decreased by 40% or more from August/September to November but had an increase from November to January were categorized as "declined/recovered". Colonies that had no honey bee covered frames remaining prior to the final sampling date were classified as "died during the monitoring period". Colonies were classified as "high *V. destructor*" if they had three or more mites per 100 bees, while "low *V. destructor*" colonies had less than three mites per 100 bees. Post-high *V. destructor* colonies were defined as those that had previously high *V. destructor* pressure but subsequently had low *V. destructor* pressure.

2.2 Honey bee sample preparation for virus analysis

Samples with the same categorical descriptions were pooled (e.g., beekeeping operation A, assessment sampling in September, high loss, high *V. destructor* pressure) for sample preparation and sequencing. In total, 38 libraries were sequenced.

For each colony-level sample, ten individual honey bees were randomly selected for analysis. Honey bees (five per 2 mL microcentrifuge tube) were homogenized in 500 µL of sterile H_2O using two sterile steel beads (4.5 mm) and a TissueLyser II (Qiagen) for 4 minutes total at 30 Hz. Bee lysates were centrifuged for two minutes at 13,500 rpm to remove debris, and supernatants were combined to yield a colony-level lysate (10 bees). A volume of 60 µL of supernatant from each colony-level lysate was pooled with lysates from colonies matching the same criteria to generate a "pooled lysate". The total volume of colony-level lysates pooled varied with the number of samples per grouping, with a range from 60-420 µL (representing one to seven colonies) (Supplementary Table S5).

2.2.1 Nuclease treatment

To enrich for viral nucleic acids, bee lysates were treated with nucleases to degrade unencapsulated (non-virion protected) DNA and RNA using 1 μL Benzonase (>=250 U) and 8 mL RNAse 1 (80 U) (Thermo) in a final volume of 500 μL buffer (20mM Tris, 1 mM $MgCl_2$, 100 mM NaCl, pH=7.6) for 1.5 hours at 37°C. Nucleases were not subjected to a heat deactivation step to prevent denaturation of virions.

2.2.2 RNA extraction

RNA was extracted from nuclease treated honey bee lysates samples using Trizol reagent (Thermo Fisher Scientific) according to manufacturer's instructions. In brief, 500 μL of Trizol was added to each sample. Samples were vortexed and incubated at room temperature for 5 minutes. Next, 150 μL of chloroform was added and each sample was mixed by inverting for 15 seconds, followed by incubation at room temperature for 2 minutes. Samples were centrifuged for 15 minutes at 12,000 x g. The upper aqueous layer (500 μL) was transferred to a 1.7 mL microcentrifuge tube and an equal volume (500 μL) of isopropanol was added to each sample, in addition to 20 μg of glycogen. Samples were precipitated at −20°C for 24 hr, then centrifuged for 10 min at 12,000 × g at 4°C. Supernatants were carefully removed by pipetting, and each pellet was washed three times with 75% ethanol and once with 100% ethanol. Ethanol was removed by decanting and pipetting following the final wash step, and RNA pellets were air dried for 10 minutes, before 15 mL of sterile H_2O was added to each sample and RNA was dissolved by vortexing. RNA quality and quantity was assessed on a Nanodrop 3000.

Additionally, to assess virus presence and abundance in colony-level samples, lysates representing individual colonies (n=10 honey bees) were not nuclease treated, and RNA was extracted using Trizol reagent. In brief, 50 μL of previously homogenized colony-level lysate was added to 250 μL of sterilized H_2O and vortexed. 300 μL of Trizol was added to each sample. Next, 100 μL of chloroform was added and each sample was mixed by inverting for 15 seconds, followed by incubation at room temperature for 2 minutes. Samples were centrifuged for 15 minutes at 12,000 x g. The upper aqueous layer (300 μL) was transferred to a 1.7 mL microcentrifuge tube, and an equal volume (300 μL) of isopropanol was added to each sample, in addition to 20 μg of glycogen. Samples were precipitated at −20°C for 24hr, then centrifuged for 10 min at 12,000 × g at 4°C. Supernatants were carefully removed by pipetting, and each pellet was washed three times with 75% ethanol and once with 100% ethanol. Ethanol was removed by decanting and pipetting following the final wash step, and RNA pellets were air dried for 10 minutes, before 15 μL of H_2O was added to each sample and RNA was dissolved by vortexing. RNA quality and quantity was assessed on a Nanodrop 3000.

2.2.2. RNA sequencing

RNA (1 μg) for each sample was sent to the Roy J. Carver Center for Biotechnology at the University of Illinois. Since samples were nuclease treated prior to RNA

extraction to enrich for viral RNA, libraries were prepared without poly-A selection or ribosomal RNA depletion using a Watchmaker RNA prep kit. RNA quality and quantity was assessed using an Agilent 3500 Fragment Analyzer. Samples were sequenced on a Novaseq X Plus 25B Flowcell (2 x 150-cycle, per lane) generating ~3.2 billion total reads. A range of 6,560,090 to 10,694,460 paired end reads (150 bp) were generated for each library, with an average read count of 8,051,730.

2.2.3 cDNA synthesis

cDNA was synthesized by incubating 2 µg of total RNA, 200 U of Moloney murine leukemia virus reverse transcriptase (M-MLV), and 500 ng of random hexamer primers in a 25 µL reaction at 37°C for 1 hour. cDNA was generated from a representative subset of RNA samples representing colony level lysates (n=10) to assess virus presence and abundance.

2.2.4 Polymerase chain reaction

PCR was performed using standard methods to screen individual colony-level lysates for partitivirus sequence presence. Briefly, 2 µL of cDNA was combined with 10 pmol of each forward and reverse primer (Supplementary Table S6). Amplification was performed using ChoiceTaq polymerase according to the manufacturer's instructions with the following conditions: 95°C for 5 minutes, 95°C for 30 s, 60°C for 30 s, 72°C for 30 s (for a total of 35 cycles) followed by a final elongation step for 5 min at 72°C. Generated PCR products were analyzed by gel electrophoresis (2% agarose with SYBR safe dye) and visualized using a Syngene U:Genius 3 imaging system. The primers used for partitivirus PCR and quantitative PCR reactions are listed in Supplementary Tables S6 and S7. To further support the *de novo* assembled undescribed virus sequences generated during this study, Sanger sequencing of an approximately 500 nt region of AmPVLC1, AmPVLC2, AmPVLR1, LSV-9, ABPV-CA-22, and Southern California dicistrovirus 1 was performed. PCR products were cleaned up using a QIAquick PCR purification kit (Qiagen) according to manufacturer's instructions. DNA quantity and quality was assessed using a Nanodrop 3000 prior to Sanger sequencing. In all cases, Sanger sequenced regions shared >99% nucleotide identity to the assembled genome.

2.2.5 Quantitative polymerase chain reaction

Quantitative PCR (qPCR) was used to analyze the abundance of AmPVLC-1, AmPVLC-2, and HPLV-34 at the colony level. qPCR reactions were performed using 2 µL of cDNA as template in triplicate. Reactions contained 1 x ChoiceTaq Mastermix, 0.2 µM of each forward and reverse primer, 1 x SYBR green, and 3 mM $MgCl_2$. Reactions were carried out in 96-well plates using a CFX Connect Real-Time instrument with the following thermo-profile: preincubation at 95°C for 1 min, followed by 40 cycles of 95°C for 10 s, 60°C for 15 s, and 72°C for 15 s. A final melt curve was generated (with

measurements at every half degree) from 65°C to 95°C for 5 s. To quantify the abundance of these sequences, plasmid standards (virus-specific qPCR amplicons cloned into pGEM-T Promega vector) for each sequence were used as templates, with a range of 10^3 or 10^4 to 10^9 copies per reaction (Supplementary Table S7. Reactions without cDNA template were used as non-template controls (NTC). The specificity of qPCR reactions was verified by melt curve analysis and gel electrophoresis. For each of the colony-level samples, the starting quantity for each well with a cDNA template representing 80 ng of total RNA was calculated using the previously generated virus-specific standard curve, and the average SQ of the NTC reaction was subtracted. Sequence abundance was reported as RNA copies (including potential genomes and transcripts) per 2 μg of total RNA. Abundance ranged from ~ 0 copies to ~ 3.8×10^{10} RNA copies/2 μg of RNA.

2.3 Bioinformatic analysis

2.3.1. Read file processing

Read files were downloaded and quality was assessed using FastQC (v.0.12.1) [4]. To remove low quality reads or possible sequencing adaptors, the trimming tool BBDuk (part of the BBtools suite) was used to trim reads (version 39.26) [5]. Example command: *reformat.sh in=$read1 in2=$read2 out=$trimmed_read1 out2=$trimmed_read2 ref=adaptors.fa ktrim=r k=23 mink=11 hdist=1 tpe tbo trimq=30*. After trimming, reads were aligned to the reference honey bee genome (NCBI Amel_HAv3.1) and the HoloBee nonviral index (an index of honey bee associated microorganisms) using HISAT2 [6–10]. Example command: *hisat2 -x amel_genome -1 $trimmed_read1 -2 $trimmed_read2 -S amel_aligned_reads.sam*. Only unmapped reads were retained using samtools [11]. Example command: *samtools -b -f 4 input_file.sam* > *unmapped.bam*. The resulting unmapped reads bam file was name sorted using samtools, example command: *samtools sort -n unmapped.bam* > *sorted_unmapped.bam*. This sorted bam file was converted to paired end FastQ files using samtools, example command: *samtools fastq sorted_unmapped.bam -1 unmapped_R1.fastq -2 unmapped_R2.fastq*.

2.3.2 De novo assembly and clustering

Paired end FastQ files containing unmapped reads were used to de novo assemble virus contigs using SPAdes (v.4.2.0), with option –rnaviral to specifically assemble RNA viruses from read files [12]. Example command: *spades.py –rnaviral -1 unmapped_R1.fastq -2 unmapped_R2.fastq -o assembly*. Assembly was performed for each individual sequencing library, generating 38 contig files (one for each library). These individual assemblies were then concatenated, generating a redundant list of contigs from all samples (i.e., duplicates of the same viral genomes). To create a nonredundant list of contigs, CD-HIT was used to cluster sequences with greater than or equal 95% nucleotide identity, and only contigs longer than 1000 nt were retained. Example

command: *cd-hit -c 0.95 -l 1000 -i redundant_contigs.fa -o nonredundant_contigs.fa*. Contigs remaining after clustering (i.e., those sharing less than 95% nucleotide similarity) were treated as unique. Initial assembly generated 152,898 contigs across all sequencing libraries. After clustering, 1,825 nonredundant contigs remained.

2.3.2 Identification of putative viral contigs

The generated list of contigs was queried against a local DIAMOND database, generated from NCBI's viral RefSeq dataset of viral proteins (release 229) in order to identify putative viral contigs [13,14]. Example command: *diamond blastx -db virus_refseq -q input_contigs.fa -o hits_out*. To identify putative viral sequences with more distant homology to known viruses, a local hidden Markov model (HMM) profile was created using the virus orthologous group (VOG) database (version 227) [15]. Open reading frames in the assembled contigs were predicted using Prodigal (version 4.3) and the corresponding translated protein sequences were queried against the VOG HMM profile using HMMscan [15,16]. Example command: *hmmscan virus.hmm predicted_proteins.faa > hmmscan_results*. Putative new virus sequences were further characterized using Geneious (version 2025.1) [17]. Across the 1,825 nonredundant contigs, 457 had homology to viruses by DIAMOND alignment, while two highly abundant contigs did not share homology with viruses by DIAMOND alignment, but did by HMMscan. Putative viral contigs were further analyzed by web-interface BLASTn, enabling the removal of false-positives and sequences appreciably shorter than the expected genome length. This resulted in a final list of 64 contigs, representing 16 well-characterized honey bee and/or *V. destructor* viruses, eight plant viruses, and 26 undescribed viruses or virus fragments.

2.3.3 Comparison of virus genome variability across sequencing libraries

To assess the variability of virus sequences between individual libraries and ensure the reported viral sequences were most representative, consensus sequences for each putative viral contig were generated for each sequencing library. This was done by trimming and filtering reads from each sequencing library, followed by alignment to the nonredundant viral contigs using HISAT2, generating a library-specific alignment for each contig. Consensus sequences were generated from these alignment files in cases where at least 1,000 reads aligned using the Geneious "Generate Consensus Sequence" function (v.2025.1). The consensus sequences for each virus were aligned using MAFFT with default parameters within Geneious [18]. The consensus sequences for each virus across sequencing libraries were very similar, sharing over 99% nucleotide identity in nearly all cases (Supplementary Table S8).

Additionally, to prevent potential biases of using a single assembled contig sequence for all alignments and verify the assembled sequences from each library were similar to one another, de novo assembly was performed using reads from each

sequencing library. This generated an assembly unique to each library. Contigs representing common bee-infecting viruses (i.e., ARV-1, ABPV, BQCV, DWV-A, DWV-B, and SBV) assembled from individual libraries were aligned to one another using MAFFT with default settings. This strategy showed that de novo assembled viral contigs from each unique sequencing library were similar, sharing >97% nucleotide identity in all cases. Thus, the virus sequences reported here were similar across libraries representing a broad geographic range and read quantification was not biased (as in, reads across libraries were similar enough to the reported sequence to not be unmapped).

2.3.4 Generation of virus consensus sequences

To generate a global consensus sequence representative of all sequencing libraries, reads from each library were independently aligned to the previously assembled non-redundant virus sequences. Each library alignment file (bam) was merged to generate a global bam using the samtools *merge* command. A pileup file of all variants was generated using the bcftools *mpileup* command. Variants were called using the bcftools *call* command. This generated a global variant call file (.vcf) representing mapped reads across libraries. A consensus sequence was generated from this global variant call file using the bcftools *consensus* command. To minimize ambiguities, the -H 1 option was used to call the majority allele at each position.

To assess if certain libraries with very high read depth were dominating the consensus sequence, resulting in a biased consensus sequence, a second strategy for consensus generation was also used. Reads from each sequencing library were aligned to the previously generated nonredundant viral contig sequences using HISAT2, generating a library-specific alignment for each viral contig. Consensus sequences were generated from these alignment files in cases where at least 1,000 reads aligned using the Geneious "Generate Consensus Sequence" function (v.2025.1). The consensus sequences for each virus were aligned using MAFFT with default parameters within Geneious [18]. This alignment file was then used to generate a final "global consensus" sequence using the Geneious "Generate Consensus Sequence" function with a threshold of 0% to minimize ambiguities (calling the most common base at each position). This technique of generating a consensus sequence for each library, followed by alignment to generate a "global" consensus representative of all libraries, was utilized to give equal weight to each library (so no single library with very high read abundance overwhelmed base calls). In cases where two bases were equally abundant at a position, an IUPAC ambiguity code was assigned. To generate a sequence with no ambiguous base calls, the sequence alignment was manually examined and the base from the sequence generated from the most reads (i.e., the consensus sequence from the library in which the virus was most abundant) was used. The consensus sequences generated

using this strategy were >99% similar to those generated using the technique described above.

2.3.5 Contig quantification and normalization

Relative contig abundances were estimated by aligning the trimmed FastQ read files to the nonredundant list of putative viral contigs with HISAT2. Example command: *hisat2 -x nr_contigs -1 $trimmed_read1_F.fq -2 $trimmed_read1_R.fq -S contig_quant_out.sam*. These alignment files were compressed to .bam files and unaligned reads were discarded using samtools. Example command: *samtools view -b -F 4 contig_quant.sam > aligned_contig_quant.bam*. These resulting .bam files were sorted using samtools. Example command: *samtools sort aligned_contig_quant.bam > sorted_aligned_contig_quant.bam*. Read counts were extracted from these alignment files using the samtools built-in *idxstats* command. Example command: *samtools idxstats sorted_aligned_contig_quant.bam > counts.txt*. To compare virus read abundance across samples, raw read counts for each virus were converted to fragment per kilobase gene per million reads (FPKM). Read counts of each trimmed FastQ file were generated using the Seqkit stats command [19]. Example command: *seqkit stats file.fastq*. Raw read counts for each virus were normalized to fragments per kilobase of transcript per million reads (FPKM). This was calculated by first dividing the read count in the trimmed FastQ file by 1,000,000, yielding the per million scaling factor for each read pair. The assembled nonredundant virus sequences aligned against were taken as transcripts in this case, and thus kilobase of transcript was calculated by dividing the genome length of a given virus by 1,000. Thus, fragments per million reads (FPM) was calculated by dividing the read count for a given virus by the per million scaling factor, and the final FPKM was calculated by dividing this FPM by the virus genome length in kilobases, thereby normalizing for sequencing depth and virus length. Top contigs with homology to known viruses were further characterized using Geneious to assess sequence similarities and predict open reading frames (ORFs). To characterize virus prevalence, a library was considered "positive" for a given virus if 1,000 or more reads aligned.

2.3.6 Relaxation of HISAT2 alignment parameters

These nonredundant viral contigs were used to create a HISAT2 index, against which reads for each sequencing library were aligned to generate coverage maps and consensus sequences. Due to the highly variable nature of RNA virus populations, HISAT2 alignment was performed with more relaxed parameters (-L, 0, -0.4) to permit a greater proportion of mismatches during alignment (~6-8 mismatches for a 150 nt read, or approximately 94.7% to 96% identity) and more reliably capture all viral reads. Testing of alignment parameters found this alignment setting, achieved with the HISAT2 parameters L, 0, -0.4 captured contigs that were not aligned under default

settings (in some instances, ~10% of contigs that did not align under default parameters did align using the relaxed parameter) (Supplementary Table S9). Further relaxing of alignment parameters did not appreciably increase the proportion of aligned contigs.

2.3.7 Assessment of read mapping specificity

To assess the specificity of read quantification by alignment to the DWV-A and DWV-B sequences, as well as the LSV2 and LSV3 sequence variants, alignment was performed using HISAT-2 with either single or multi mapping enabled. Differences in read counts for DWV-A, DWV-B, and the LSV variants using single or multimapping were negligible, indicating reads were not aligning to both DWV-A and DWV-B or to multiple LSV variants (Supplementary Table S10).

REFERENCES

1. Glenny, W.; Cavigli, I.; Daughenbaugh, K.F.; Radford, R.; Kegley, S.E.; Flenniken, M.L. Honey bee (Apis mellifera) colony health and pathogen composition in migratory beekeeping operations involved in California almond pollination. *PLoS ONE* **2017**, *12*, e0182814, doi:10.1371/journal.pone.0182814.
2. Sagili, R.R.; Burgett, D.M. Evaluating Honey Bee Colonies for Pollination. Pac. Northwest Ext. **2011**, 623. https://api.semanticscholar.org/CorpusID:81067252.
3. Faurot-Daniels, C.; Glenny, W.; Daughenbaugh, K.F.; McMenamin, A.J.; Burkle, L.A.; Flenniken, M.L. Longitudinal monitoring of honey bee colonies reveals dynamic nature of virus abundance and indicates a negative impact of Lake Sinai virus 2 on colony health. *PLOS ONE* **2020**, *15*, e0237544, https://doi.org/10.1371/journal.pone.0237544.
4. Andrews, S. FastQC: A Quality Control Tool for High Throughput Sequence Data **2010**.
5. Bushnell, B. BBMap: A Fast, Accurate, Splice-Aware Aligner. **2014**.
6. Elsik, C.G.; Worley, K.C.; Bennett, A.K.; Beye, M.; Camara, F.; Childers, C.P.; de Graaf, D.C.; Debyser, G.; Deng, J.; Devreese, B.; et al. Finding the missing honey bee genes: lessons learned from a genome upgrade. *BMC Genom.* **2014**, *15*, 1–29, https://doi.org/10.1186/1471-2164-15-86.
7. Weinstock, G.M.; Robinson, G.E.; Gibbs, R.A.; Weinstock, G.M.; Weinstock, G.M.; Robinson, G.E.; Worley, K.C.; Evans, J.D.; Maleszka, R.; Robertson, H.M., et al. Insights into social insects from the genome of the honeybee Apis mellifera. *Nature* **2006**, *443*, 931–949, doi:10.1038/nature05260.
8. Evans, J.; Schwarz, R.; Childers, A. HoloBee Database V2016.1. **2016**.
9. Kim, D.; Paggi, J.M.; Park, C.; Bennett, C.; Salzberg, S.L. Graph-based genome alignment and genotyping with HISAT2 and HISAT-genotype. *Nat. Biotechnol.* **2019**, *37*, 907–915, https://doi.org/10.1038/s41587-019-0201-4.
10. Kim, D.; Langmead, B.; Salzberg, S.L. HISAT: A fast spliced aligner with low memory requirements. *Nat. Methods* **2015**, *12*, 357–360, doi:10.1038/nmeth.3317.
11. Li, H., Handsaker, B., Wysoker, A., Fennell, T., Ruan, J., Homer, N. The Sequence Alignment/Map format and SAMtools. *Bioinformatics* **2009**, *25*, 2078–2079, https://doi.org/10.1093/bioinformatics/btp352.
12. Antipov, D.; Raiko, M.; Lapidus, A.; A Pevzner, P. Metaviral SPAdes: assembly of viruses from metagenomic data. *Bioinformatics* **2020**, *36*, 4126–4129, https://doi.org/10.1093/bioinformatics/btaa490.
13. Buchfink, B.; Xie, C.; Huson, D.H. Fast and sensitive protein alignment using DIAMOND. *Nat. Methods* **2015**, *12*, 59–60, https://doi.org/10.1038/nmeth.3176.

14. Brister, J.R.; Ako-Adjei, D.; Bao, Y.; Blinkova, O. NCBI Viral Genomes Resource. *Nucleic Acids Res.* **2014**, *43*, D571–D577, https://doi.org/10.1093/nar/gku1207.
15. Eddy, S. HMMER: Biosequence Analysis Using Profile Hidden Markov Models **2023**.
16. Hyatt, D.; Chen, G.-L.; Locascio, P.F.; Land, M.L.; Larimer, F.W.; Hauser, L.J. Prodigal: prokaryotic gene recognition and translation initiation site identification. *BMC Bioinform.* **2010**, *11*, 119. https://doi.org/10.1186/1471-2105-11-119.
17. Geneious Prime 2025.
18. Katoh, K.; Standley, D.M. MAFFT Multiple Sequence Alignment Software Version 7: Improvements in Performance and Usability. *Mol. Biol. Evol.* **2013**, *30*, 772–780, https://doi.org/10.1093/molbev/mst010.
19. Shen, W.; Le, S.; Li, Y.; Hu, F. SeqKit: A Cross-Platform and Ultrafast Toolkit for FASTA/Q File Manipulation. *PLOS ONE* **2016**, *11*, e0163962, https://doi.org/10.1371/journal.pone.0163962.
