# Supplementary material for: Honey Bee Viromes from Beekeeping Operations Experiencing High Losses in 2022–2023
Source: Viruses. 2026 Mar 9;18(3):334. doi: 10.3390/v18030334 (PMC13030351; doi:10.3390/v18030334)

Figure S1. Acute bee paralysis (ABPV-CA-22) variant

(A) Schematic of acute bee paralysis virus sequence variant identified from honey bee samples obtained in 2022-2023 from California-based commercial beekeeping operations, ABPV-CA-22 (~ 9,660 nt, GenBank PX726311). At the nucleotide level, ABPV-CA-22 is 90.8% identical to ABPV isolate No11_Am029-XJ2018 (GenBank MZ821781). The even distribution of the nucleotide differences (black lines) suggest that ABPV-CA-22 is a genetic variant and not a recombinant strain.
(B) The ABPV-CA-22 genome was assembled from short read sequencing data, the coverage map (blue shaded region) generated from mapping reads from library 24 to the ABPV-CA-22 genome had a maximum coverage of 8,263x and an average coverage of 5,168x, and matched genome sequence. In addition, genome assembly and sequences were supported by Sanger sequencing of ~ 500 nt of the ABPV-CA-22 genome (region ~4,500 – 5,000 nt); this region was >99% identical to the reported genome (gray bar, nuclelotide differences denoted by black lines).
(C) ABPV-CA-22 has two predicted open reading frames (ORFs) that encode a nonstructural polyprotein that includes the RNA-dependent-RNA polymerase (RdRp), and a structural polyprotein that encodes the capsid proteins. These polyproteins are 95.3% and 95.7% identical with the amino acid sequence of the previously reported ABPV isolate No11_Am029-XJ2018.

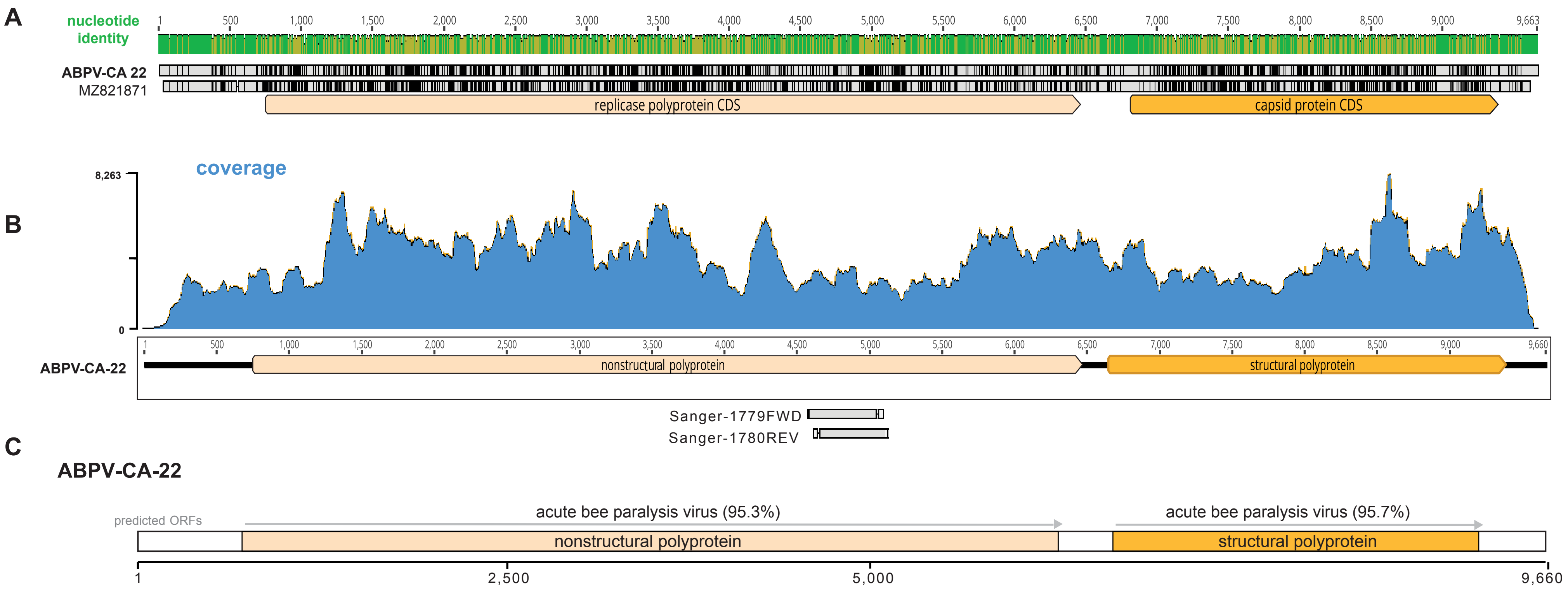

Figure S2. Lake Sinai virus 9

(A) Schematic of Lake Sinai virus-9 (LSV-9), a sequence variant identified from honey bee samples obtained in 2022-2023 from California-based commercial beekeeping operations, LSV-9 (~ 6,065 nt, GenBank PX726310). At the nucleotide level, LSV-9 is 83.7% identical to LSV-8 Goryeong (GenBank OR496495). The even distribution of the nucleotide differences (black lines) suggests that LSV-9 is a genetic variant and not a recombinant strain.
(B) The LSV-9 genome was assembled from short read sequencing data, the coverage map (blue shaded region) generated from mapping reads from library 21 to the LSV-9 genome had a maximum coverage of 170,730x and an average coverage of 97,287x and matched the genome sequence. In addition, genome assembly and sequences were supported by Sanger sequencing of ~ 500 nt of the LSV-9 genome (region ~500 – 1,000 nt); this region was >99% identical to the reported genome (gray bar, nuclelotide differences denoted by black lines).
(C) LSV-9 has three predicted ORFs that encode a nonstructural polyprotein, an RdRp, and a capsid protein. These polyproteins are 87.9%, 90.2%, and 95.6% identical with the amino acid sequence of LSV-8 Goryeong.

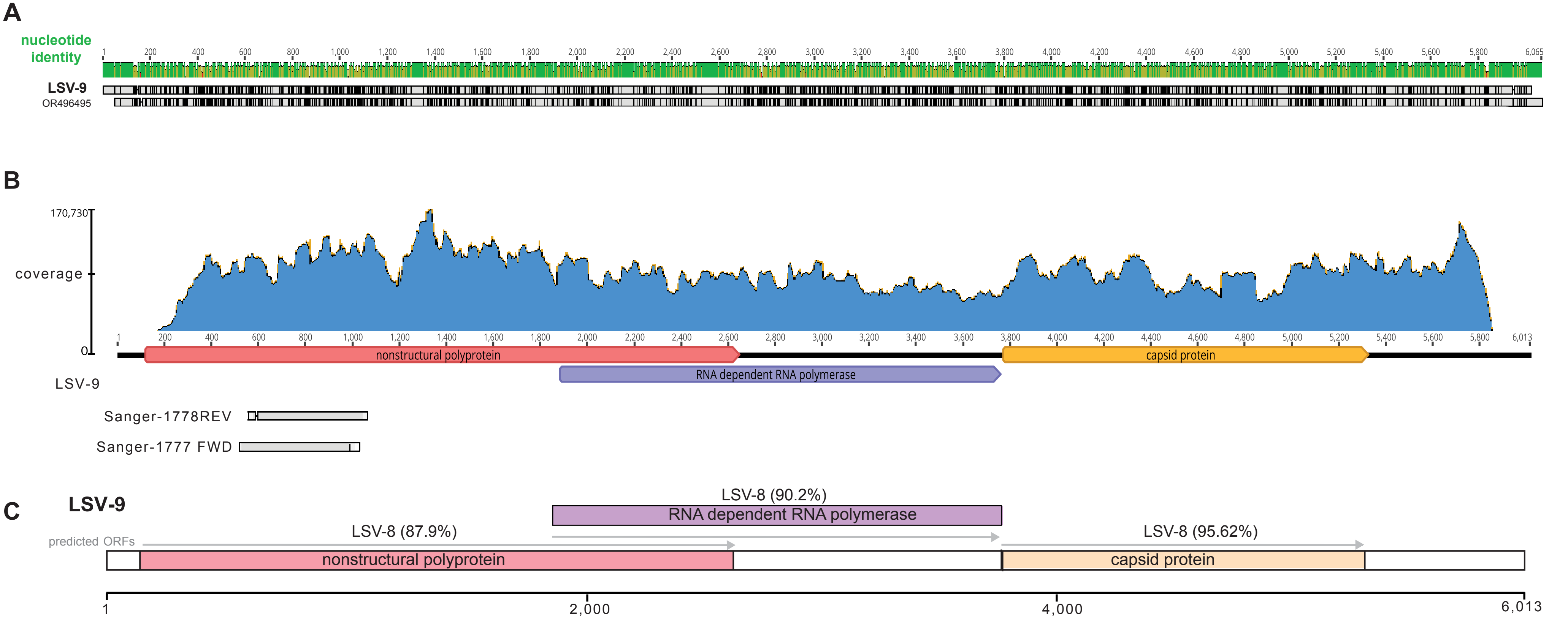

Figure S3. Partitivirus like genome schematics

Sequencing schematic of five partiti-like virus segments found in this study, including two that that were previously reported (A. mellifera partiti-like virus 1 (AmPLV1–PV664202) and Hubei partiti-like virus 34 (HPLV_34–OR496448)) and three discovered in this study (A. mellifera partiti-like virus RdRp 1 (AmPVLR-1– PX726309, A. mellifera partitivirus like capsid 1 (AmPVLC-1– PX726307), and A. mellifera partitivirus like capsid 2 (AmPVLC-2– PX726308). Predicted ORFs and putative encoded proteins are illustrated. Homologous proteins were identified using BLASTp with the most homologous protein listed with the percent amino acid identity. For AmPVLC-1 and AmPVLC-2 BLASTp analyses produced no results, thus, AlphaFold2 was used to predict protein structure to query Foldseek; the top result is listed with the associated E-value.

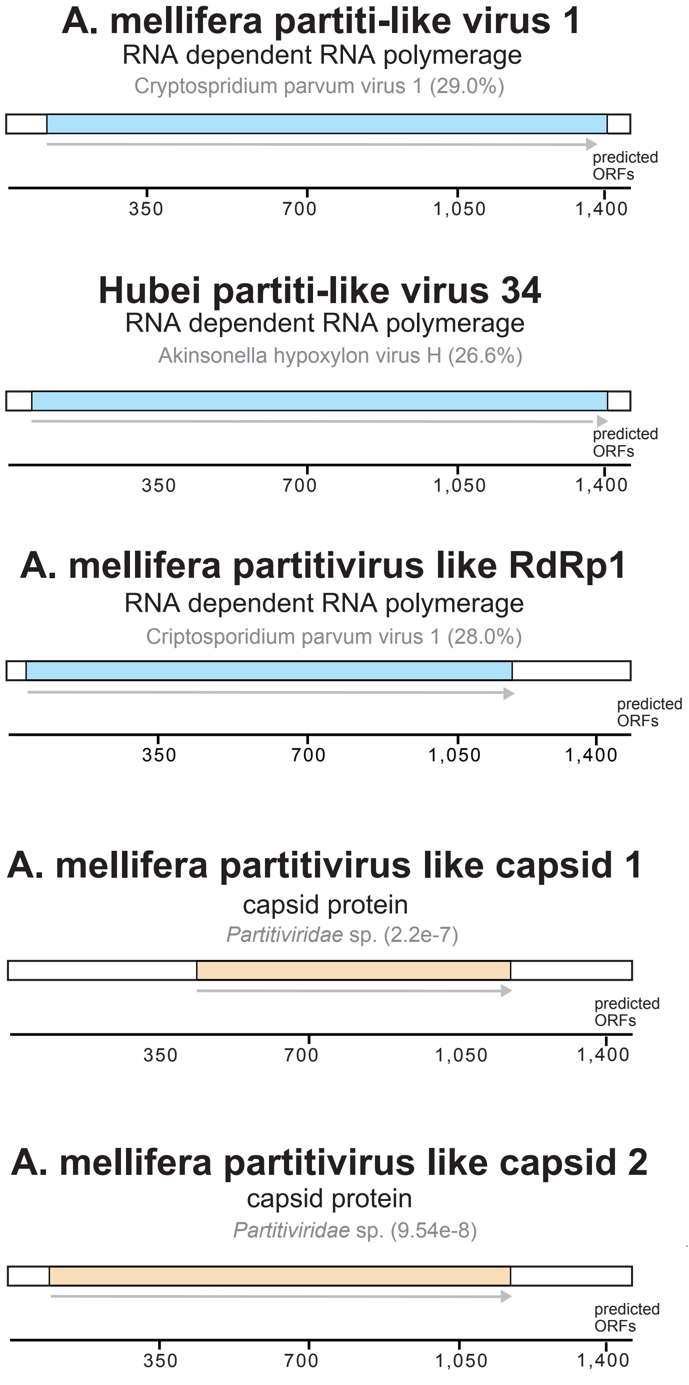

Figure S4. AmPVLC-1, AmPVLC-2, and AmPVLR-1 abundance was positively correlated in sequence libraries

The abundances (FPKM) of AmPVLC-1, AmPVLC-2, and HPLV-34 were positively correlated at the sequencing library level. These correlations were strong (R^2=0.99 for AmPVLC-1 and AmPVLC-2, R^2=0.89 for HPLV-34 and AmPVLC-1, R^2=0.89 for HPLV-34 and AmPVLC-2, and R^2=0.99 for AmPVLC-1, AmPVLC-2, and HPLV-34; all Spearman's correlation). These correlations suggest the sequences may comprise segments of the same multipartite partitivirus genome.

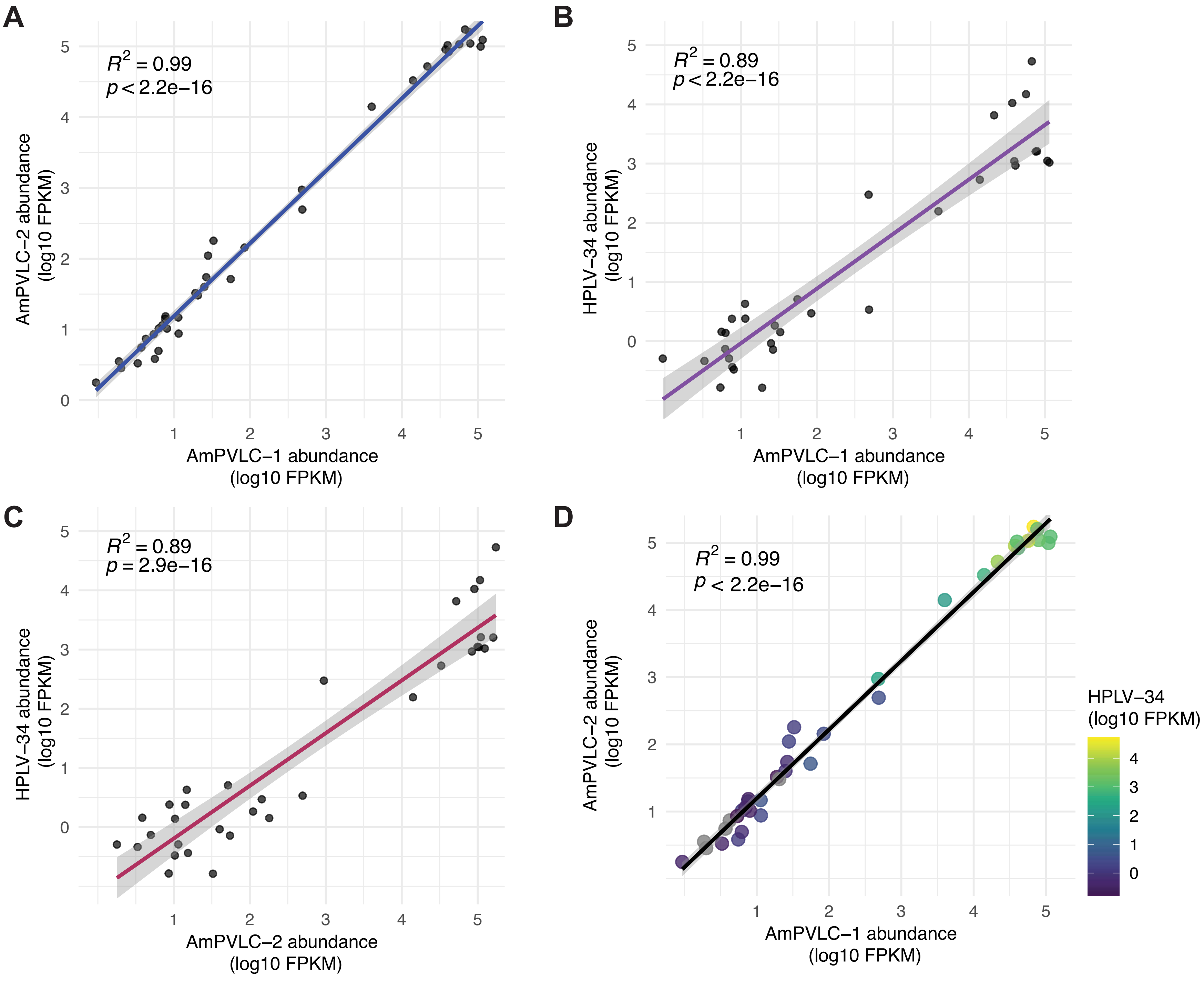

Figure S5. Co-occurrence of AmPVLC-1, AmPVLC-2, and AmPVLR-1 at the colony level

Partitiviruses are multipartite dsRNA viruses. Polymerase chain reaction was used to examine the potential co-occurrence of the partiviruses that were prevalent and abundant in this sample cohort (i.e., AmPVLC-1, AmPVLC-2, HPLV-34, AmPLV1, and AmPVLR-1) at the colony level. Individual colony level samples (n=29) were selected from the sample lists for sequencing libraries that had high partitivirus abundance (>500,000 reads). AmPVLC-1, AmPVLC-2, and HPLV-34 were commonly detected in the same samples; their co-occurrence supports the hypothesis that these sequences are segments of a single partitivirus genome.

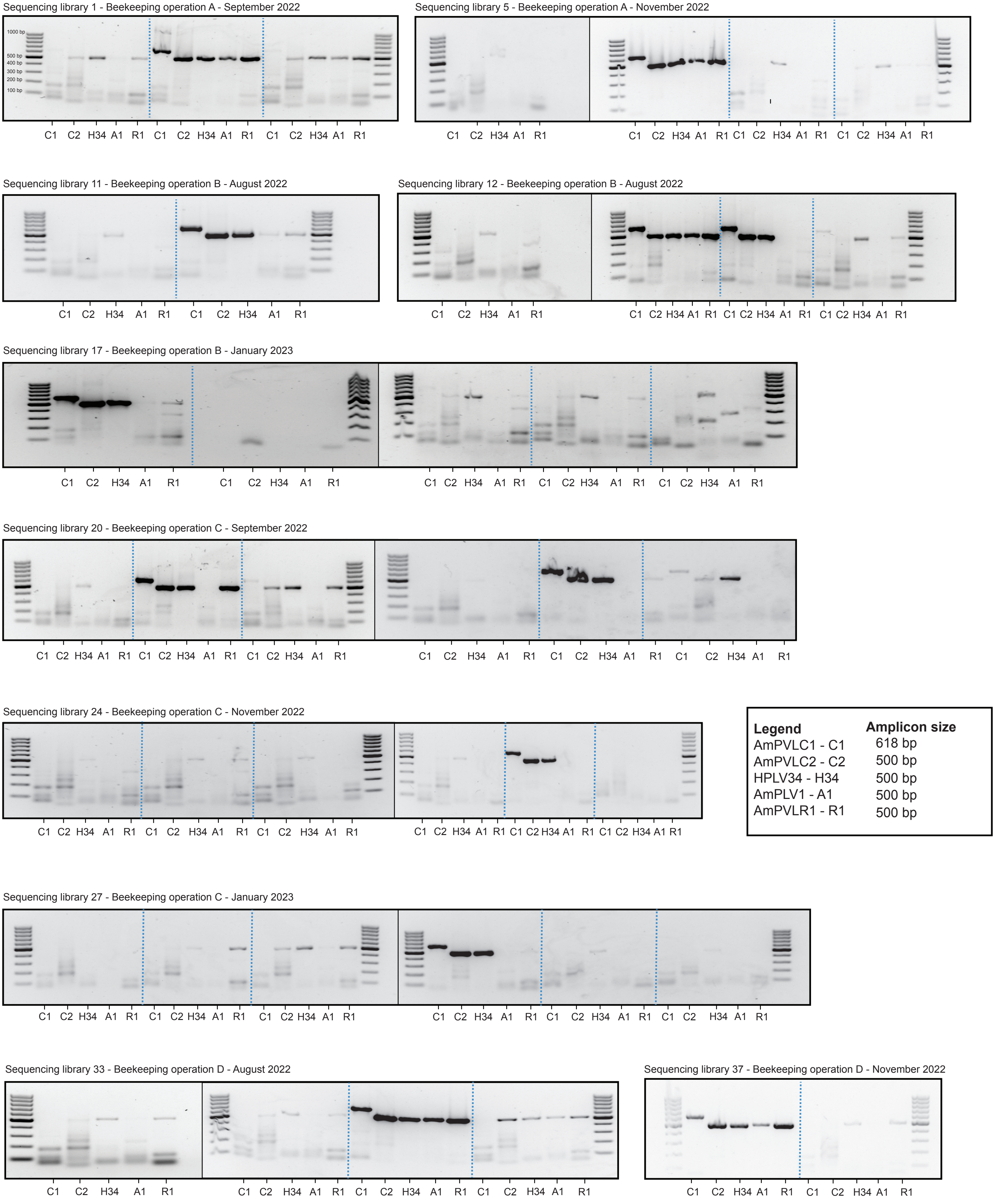

Figure S6. Abundance of AmPVLC-1, AmPVLC-2, and HPLV-34 correlates at the colony level

To assess the correlation of AmPVLC-1, AmPVLC-2, and HPLV-34 abundance at the colony level and validate the abundance data observed for each sequencing library (Appendix Figure 4), qPCR was performed on colony level samples (n=43). Pairwise analyses indicated that abundance of these sequences were highly correlated (Spearman's correlation).
(A) AmPVLC1 and AmPVLC2, $R^2 = 0.97$, (B) AmPVLC1 and HPLV-34, $R^2 = 0.93$, (C) AmPVLC2 and HPLV-34, $R^2 = 0.95$, and (D) $R^2 = 0.97$ when all three segments were compared. This trend supported the quantification data seen in each sequencing library. This similarity between the trends observed in partitivirus abundance at the sequencing library level (by FPKM) and individual colony-level (by qPCR, SQ values) supports the sequencing data and quantification of read abundance by FPKM.

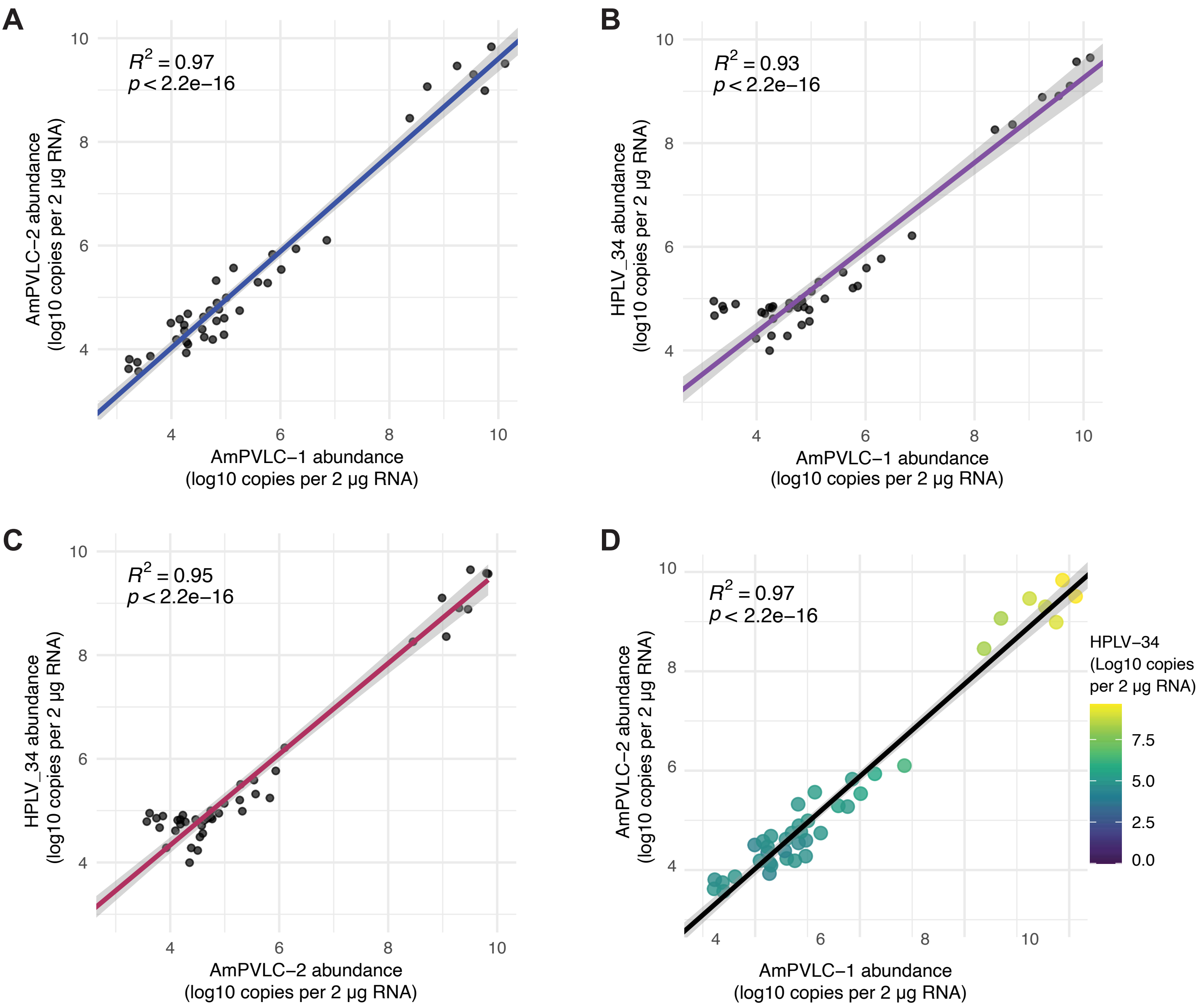

Figure S7. Two unclassified contigs correlated with partitivirus sequence abundance

Two undescribed contigs were highly abundant in sequencing libraries and were significantly correlated with partitivirus like sequence abundance. Specifically, unknown abundant contig 1 correlated with AmPVLC-1, AmPVLC-2, and HPLV-34 (R^2= 0.90, 0.98, and 0.96, respectively, Spearman's correlation) and unknown abundant contig 2 was correlated with AmPLV1 and AmPVLR-1 (R^2 = 0.79 and 0.83, respectively, Spearman's correlation).

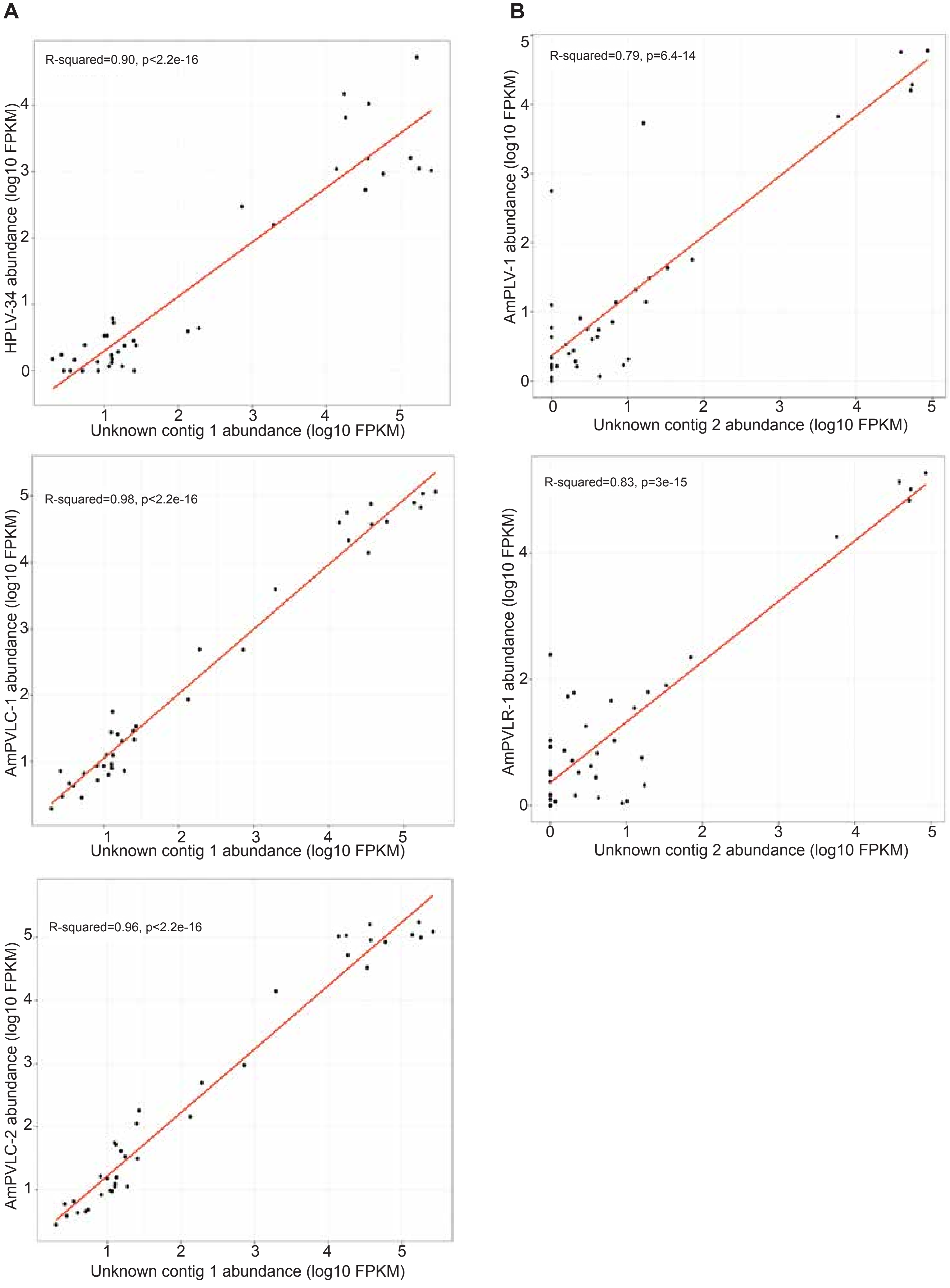

Figure S8: Virus abundance in sequencing libraries from August & September 2022

Individual sequencing libraries for each given assessment timepoint. The five most abundant viruses are graphed per library as separate bars on the x-axis; the y-axis gives normalized read abundance (Log10(FPKM+1)) per library.

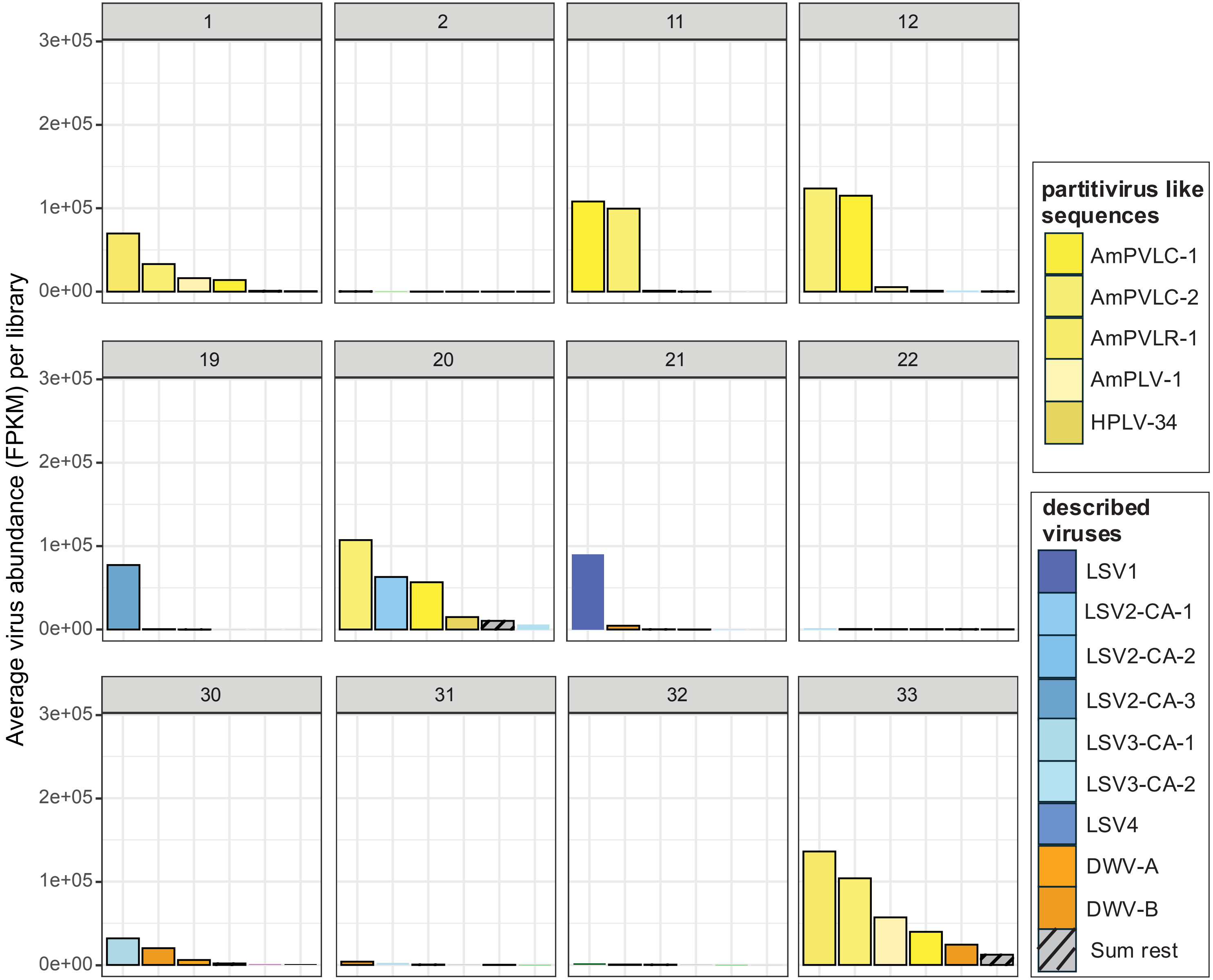

Figure S9: Virus abundance in sequencing libraries from November 2022

Individual sequencing libraries for each given assessment timepoint. The five most abundant viruses are graphed per library as separate bars on the x-axis; the y-axis gives normalized read abundance (Log10(FPKM+1)) per library.

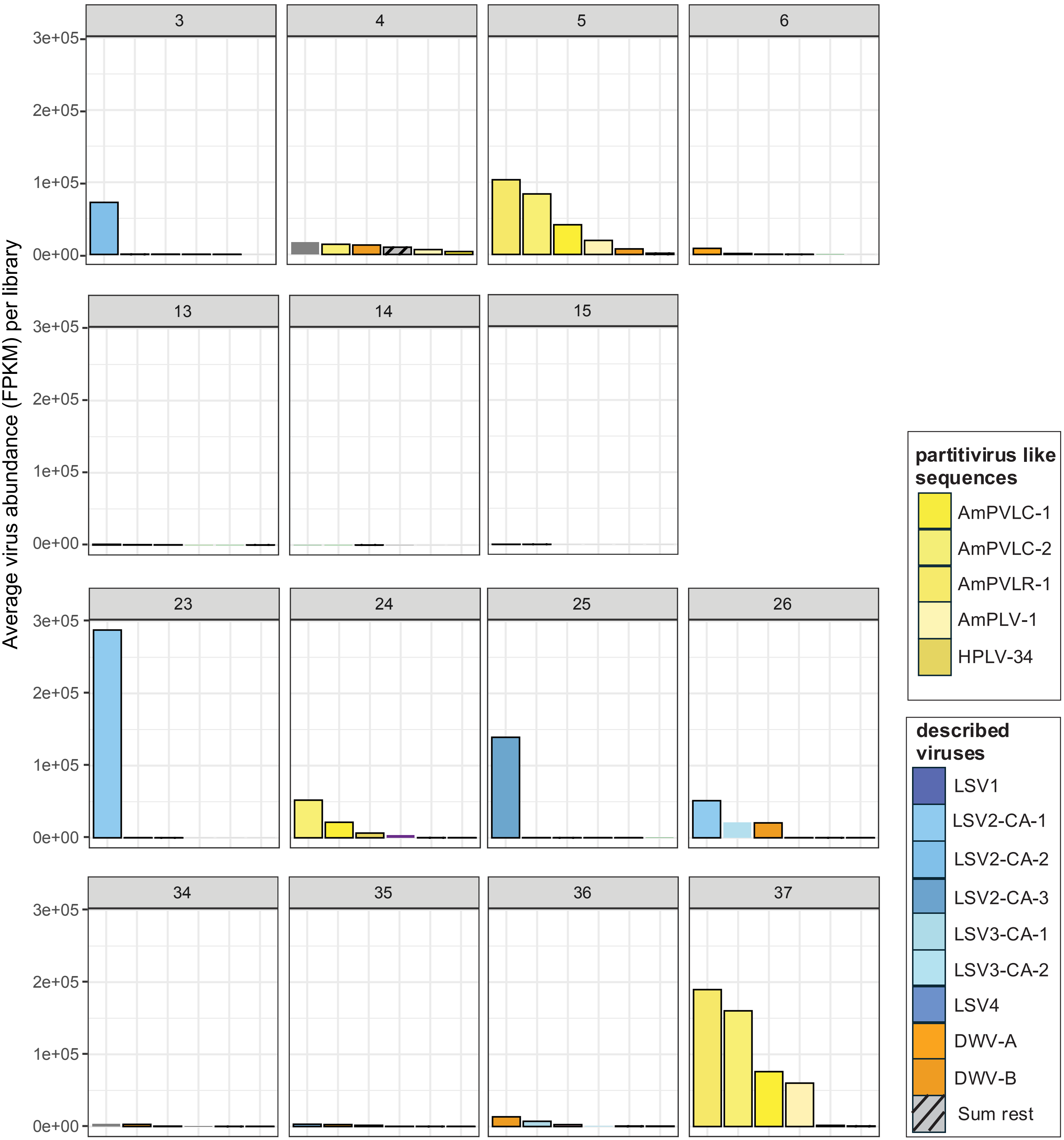

Figure S10: Virus abundance in sequencing libraries from January 20223

Individual sequencing libraries for each given assessment time point. The five most abundant viruses are graphed per library as separate bars on the x-axis; the y-axis gives normalized read abundance (Log10(FPKM+1)) per library.

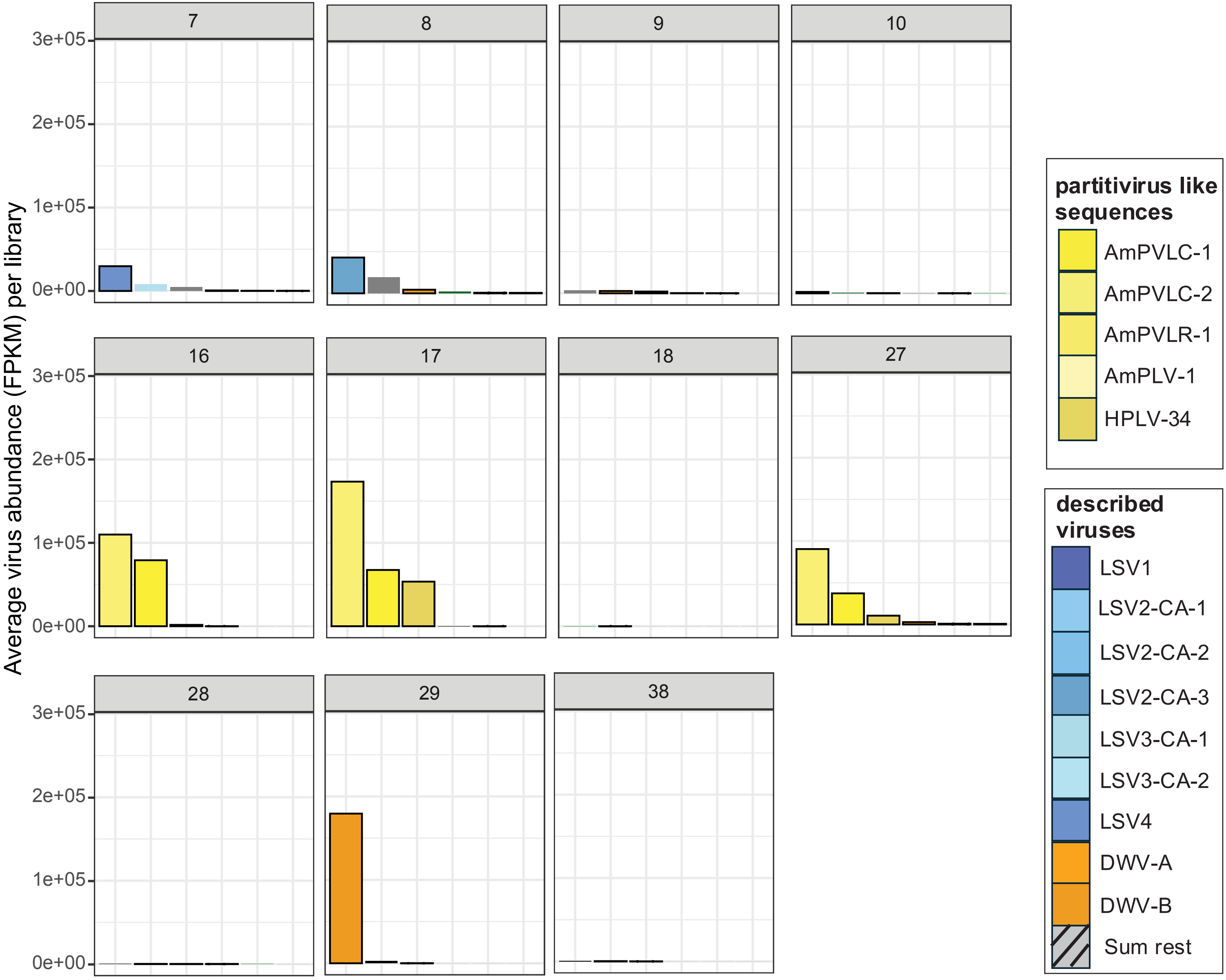

Supplement: Supplementary file 1 [file viruses-18-00334-s001.zip › Supplementary_Figures_Merged_SFigs.pdf]
